# Microprotein-encoding RNA regulation in cells treated with pro-inflammatory and pro-fibrotic stimuli

**DOI:** 10.1186/s12864-024-10948-1

**Published:** 2024-11-05

**Authors:** Victor J. Pai, Calvin J. Lau, Almudena Garcia-Ruiz, Cynthia Donaldson, Joan M. Vaughan, Brendan Miller, Eduardo V. De Souza, Antonio M. Pinto, Jolene Diedrich, Narender R. Gavva, Shan Yu, Christopher DeBoever, Shane R. Horman, Alan Saghatelian

**Affiliations:** 1 Clayton Foundation Peptide Biology Laboratories, The Salk Institute for Biological Studies, 10010 North Torrey Pines Road, La Jolla, CA 92037 USA; 2 Mass Spectrometry Core, The Salk Institute for Biological Studies, 10010 North Torrey Pines Road, La Jolla, CA 92037 USA; 3 Takeda Development Center Americas, Inc, San Diego, CA 92121 USA

**Keywords:** Small open reading frames (smORFs), Microproteins, Ribosome profiling, Inflammation, Fibrosis

## Abstract

**Background:**

Recent analysis of the human proteome via proteogenomics and ribosome profiling of the transcriptome revealed the existence of thousands of previously unannotated microprotein-coding small open reading frames (smORFs). Most functional microproteins were chosen for characterization because of their evolutionary conservation. However, one example of a non-conserved immunomodulatory microprotein in mice suggests that strict sequence conservation misses some intriguing microproteins.

**Results:**

We examine the ability of gene regulation to identify human microproteins with potential roles in inflammation or fibrosis of the intestine. To do this, we collected ribosome profiling data of intestinal cell lines and peripheral blood mononuclear cells and used gene expression of microprotein-encoding transcripts to identify strongly regulated microproteins, including several examples of microproteins that are only conserved with primates.

**Conclusion:**

This approach reveals a number of new microproteins worthy of additional functional characterization and provides a dataset that can be queried in different ways to find additional gut microproteins of interest.

**Supplementary Information:**

The online version contains supplementary material available at 10.1186/s12864-024-10948-1.

## Introduction

The completion of genome projects provided a way to determine the composition of proteomes via algorithmic prediction. However, the rules underlying these predictions were predicated on known proteins and had a blind spot for small proteins, or microproteins, encoded by small open reading frames (smORFs < 100–150 codons) [1–5]. smORFs were ignored by these algorithms because random assembly of a genome from 63 codons would also result in many random smORFs that are unlikely to be functional [6–8]. Additionally, most smORFs and microproteins are not conserved, which is incongruent with typical assumptions of function [6–8], though this assumption is up for debate as new analyses reveal the prevalence of de novo gene birth as a means to obtain functional lineage specific smORFs [9–13]. These challenges mean that most microproteins are omitted from current builds of our genomic and proteomic databases [14].

Interest in smORFs and microproteins reemerged with the development and application of new detection methods. For instance, advancement in proteogenomics, which combine proteomics with RNA-Seq data to enable the detection of both known and unannotated protein-coding genes [15], has led to the discovery of hundreds of microproteins in different proteomes [16]. More broadly, the search for protein-coding ORFs, including smORFs, was bolstered by the advent of an ingenious method called ribosome profiling, or Ribo-Seq [17, 18]. Ribo-Seq identifies the regions of transcriptome bound to translating ribosomes and in doing so provides a snapshot of cellular translation that can be used to identify thousands of novel translated smORFs. Together these methods have led to the discovery that there exist thousands of unannotated protein-coding genes, mostly microproteins. Even if a small percentage of these genes are biologically active, it could still mean there are hundreds of functional microproteins that could hold the key for understanding homeostatic and disease mechanisms. Furthermore, many of these microproteins may be biologically important and have functional roles that are outside our classical definition of a functional protein. For example, many microproteins might be short lived but can have a function because they are processed and presented as HLA epitopes for immune cell signaling [19, 20]. Microproteins can also provide a reservoir of evolvable loci [21, 22] and the smORFs that encode microproteins can have regulatory roles in translation [23].

Evolutionary conservation has proven to be the most reliable method for identifying functional microproteins. Conservation can be determined using algorithms such as PhyloCSF or tBLASTn, with proteins that are identical or close to identical being ideal candidates for further characterization [24, 25]. For example, the microprotein CYREN which is conserved from mouse to human and was first detected during a proteogenomics experiment [26], was later found to control DNA repair pathway choice during the cell cycle [27, 28]. Using evolutionary conservation also led to the discovery of a family of microproteins in muscle that regulate calcium flux in the ER [29–31]. One of these muscle microproteins is called DWORF and was shown to regulate muscle function by modulating the function of its interacting protein, Sarcoendoplasmic Reticulum Calcium ATPase (SERCA), to control calcium dynamics in the muscle. Moreover, in vivo studies revealed that overexpression of DWORF can ameliorate disease outcome in a genetic mouse model of dilated cardiomyopathy [31, 32].

Conservation across species has been a reliable method for identifying functional smORFs, but evidence shows that the degree of conservation can vary significantly. Some smORFs are conserved only among vertebrates, such as the mitochondrial microprotein BRAWNIN [33], while others are unique to a single species, like those found in yeast [34]. This variability is particularly evident in the immune system, where species-specific smORFs may play unique roles that are not conserved across different organisms. For example, a recent study that looked for novel translation products during inflammation in mice identified a microprotein which was derived from the Aw112010 RNA, which prior to this work was thought to be non-coding, as a key regulator of mucosal immunity [35]. Specifically, mice that lack this microprotein were unable to control salmonella infection as well as wild-type controls. Thus, Aw112010 is a key gene in mice that is necessary for proper functioning of the gut mucosal immunity but this microprotein is not conserved in humans. Thus, it is likely like human- or primate- specific immune regulating microproteins exist but have yet to be identified.

This example suggests that strict conservation requirements might miss interesting microproteins and suggests that leveraging gene expression data might be a valuable approach for revealing functional, yet non-conserved microproteins. Here, we test this idea by using intestinal cell lines, primary cells, and tissues to determine if we can find gut microproteins that are regulated by inflammatory or fibrosis-triggering stimuli. We chose the gut because it is a remarkable tissue that serves as an essential barrier for health, transporter of nutrients for life, critical endocrine tissue, and home to our microbiomes [36]. Between endocrine peptides such as GLP-1 and others [37] and microbiome-regulating antimicrobial peptides [38, 39], the gut is also home to critically important peptides and small proteins. In this work, we have generated a dataset of intestinal and PBMC microproteins via ribosome profiling of several human intestinal cell lines and PBMCs under different conditions. More specifically, to profile human colon epithelial cells in vitro, we used Caco-2 and HCEC-1CT. Caco-2 is the most commonly studied intestinal epithelial cell line derived from human colon adenocarcinoma [40, 41]. To compare Caco-2 against a non-malignant intestinal epithelial cell line, we used HCEC-1CT, which is derived from normal human colon biopsies [42]. To profile human colon fibroblasts, we used CCD-18Co, a normal colon fibroblast line that is commonly used to assay intestinal fibrosis in vitro [43]. Finally, we profiled PBMCs to assay microprotein expression after stimulation with either LPS (activating monocytes/macrophages) or anti-CD3/CD28 (activating T-cells). The resulting Ribo-Seq dataset identified thousands of microprotein-coding genes and is one of the largest sets of microproteins determined to date, which is likely due to the diversity of cells used in this study. Of these, we found ~ 1500 of these microproteins are conserved between mice and human. However, we also find many strongly regulated microproteins are primate specific that are regulated by inflammatory stimuli (in vitro) or in disease, as determined by looking at RNA-Seq data from these conditions. Together, the data support the hypothesis that there exist tens to hundreds of biologically interesting and disease-regulated microproteins that might be markers for disease and after further study identify a role in disease.

## Results

### Overall experimental strategy and assembly of initial smORF dataset

We carried out Ribo-Seq experiments on intestinal cell lines and PBMCs to build a microprotein dataset. While Ribo-Seq can be carried out in tissues, cells provide a more tractable system for annotating smORFs that are present in normal, pro-inflammatory, or pro-fibrotic conditions. We selected several commonly used colon epithelial cell lines (Caco2, HCEC-1CT, CCD18-Co), and also included peripheral blood mononuclear cells (PBMCs) since activated immune cells, such as macrophages, monocytes, and dendritic cells, are expected to be present in the gut during inflammation [44, 45].

**Fig. 1:**
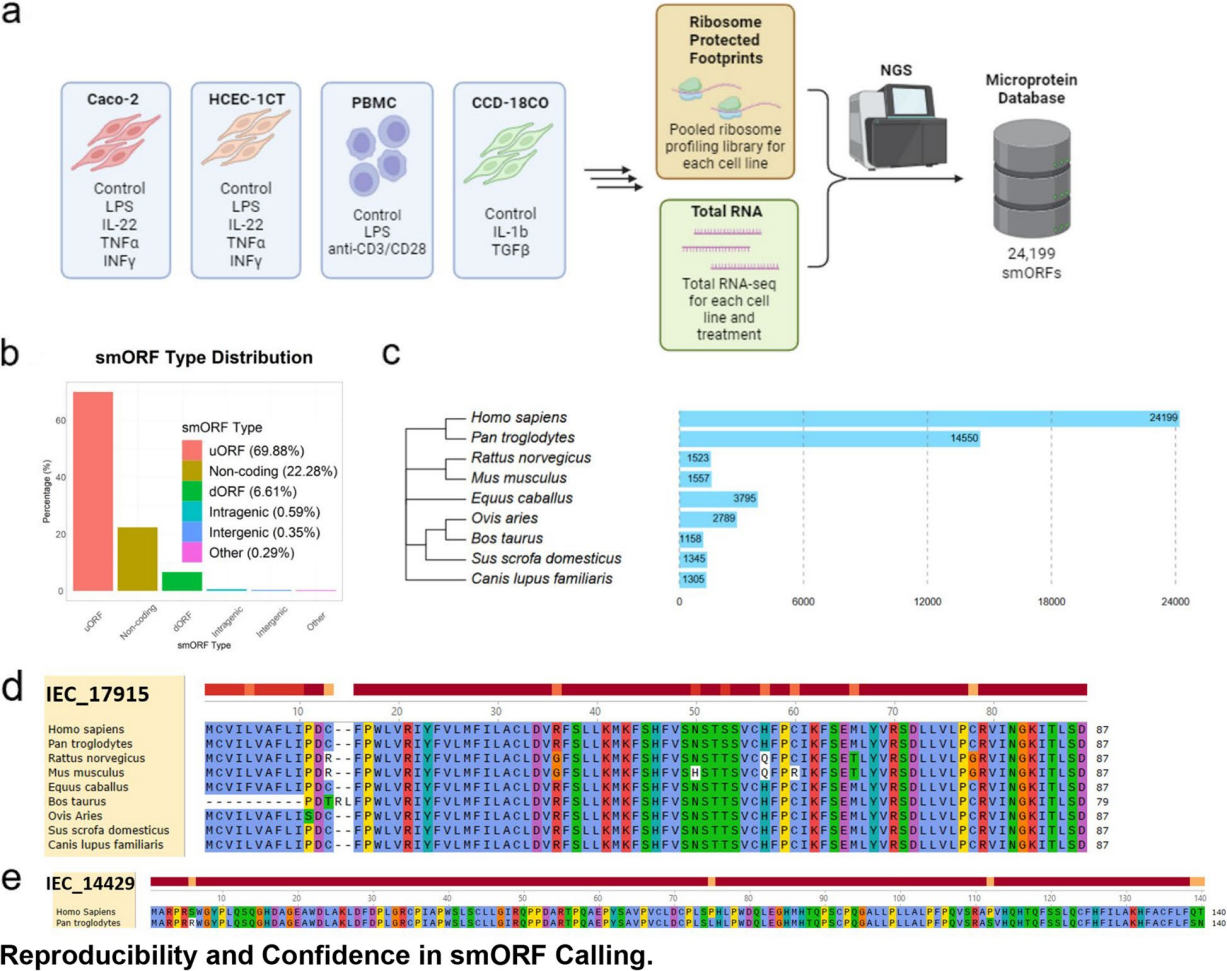
Annotation of novel smORFs from intestinal epithelial cells. **a **Overall experimental outline. Briefly, cells were treated with various stimuli as shown. Treated cell lines were pooled together for ribosome profiling or individually processed for RNA-sequencing. Altogether, 24,199 new novel smORFs were identified. **b** Distribution of smORFs across the HG38 reference genome, analyzed by location using “bedtools closest” function. uORF = upstream ORF or upstream of transcriptional start site. dORF = downstream ORF or downstream of transcriptional termination site. **c** Unrooted phylogenic tree inferred from mitochondria DNA of closely related species. Bars indicate numbers of homologous sequences for novel smORFs that are found in the genome of represented species as predicted by tBLASTn. **d**, **e** Amino acid level conservation of predicted smORFs IEC_17915 and IEC_14429

To probe clinically relevant microproteins that are specific to certain cellular phenotypes, cells were treated with a variety of different stimulants to ensure that any smORFs present within transcripts expressed under specific conditions are also detected (Fig. 1a). We focused these treatment conditions primarily on inflammatory conditions, but also included TGFβ for as a pro-fibrotic stimulant. In these experiments Caco-2 and HCEC-1CT cells were treated with several pro-inflammatory molecules including the TLR4 ligand LPS, the cytokine IL-22, TNFα, and IFNγ (Fig. 1a). PMBCs were treated with LPS and an anti-CD3/CD28 antibody which activates T cells. CCD18-Co cells were treated with IL-1b, TNFα, and TGFβ which induces a pro-fibrotic phenotype in intestinal epithelial cells (Fig. 1a). We combined and analyzed the Ribo-Seq data for all the cell lines and treatment conditions to generate a smORF dataset.

These RiboSeq data were processed and analyzed by our previously established bioinformatic pipeline that we modified to allow smORF mapping to the hg38 genome. This analysis identified a total of 24,199 unannotated intestinal epithelial cell (IEC) smORFs among the four different cell lines (Supplementary Table S1). Here, unannotated is defined as a smORF that is absent from Genbank and UniProt databases. Unannotated IEC smORFs were defined as those smORFs producing microproteins that gave no hits in BLASTp searches against the human reference non-redundant protein sequences. Note that our bioinformatic pipeline utilizes RibORF to annotate smORFs [46]. Although other tools are available for annotating ORFs in RiboSeq data, comparisons between these tools have shown poor overlap when identifying unannotated smORFs [47]. As a community resource, we incorporated two additional RiboSeq annotators, PRICE [48] and Ribocode [49], to identify further candidate smORFs (Supplementary Table S1). As expected, only 2,962 (~ 12%) of IEC smORFs were identified by more than one annotation tool, and 610 (~ 2.5%) were annotated by all three (Figure S1a). For this study, we primarily focused on smORFs identified by RibORF, as setting up and optimizing each bioinformatic pipeline requires significant effort and adjustments and while the number and smORFs differ between the different callers the overall conclusions we obtain from our analysis should not be changed.

QC-analysis of our Ribo-Seq data reveals clear 3-nt periodicity (Figure S1b, c). The median IEC smORF length is 31 codons (amino acids in the microprotein), and frequency distribution analysis results in a fitted decay curve with a constant of ~ 0.02 (Figure S1d). Furthermore, an analysis of amino acid frequency among these smORFs reveals enrichment in alanine, glycine, proline, and arginine (Figure S1e). Finally, ~ 70% of IEC smORFs overlapped with 5’UTRs of annotated genes, ~ 22% overlapped with non-coding RNAs, ~ 7% overlapped with 3’UTRs of annotated genes, and less than 1% overlapped with inter/intragenic regions of the genome (Fig. 1b). These characteristics are consistent with what is routinely seen in the literature for mammalian smORFs [20, 50].

Conservation has been the most commonly used proxy for function with smORFs, and as a consequence most of the functionally characterized microproteins are conserved. To estimate what percentage of these IEC smORFs might be functional, we employed tBlastn to find similar sequences within the genomes of various other species (Figure 1c, Supplementary Table S2). Not surprisingly, we found 14,550 (or ~60%) of IEC smORFs are conserved between *Pan Troglodytes *and *Homo *Sapiens as determined by their passing of the scoring threshold of E<0.001. Overlap with other species ranged from 1158 to 3795 (or 4.7% to 15.6%) smORFs. For example, 1557 (or 6.4%) IEC smORFs between human and mouse show high similarity and at this evolutionary distance the resulting microproteins from these smORFs are very likely to have biochemical, cellular, or physiological functions. As the field has found microprotein conservation to be an ideal surrogate for function, we hypothesize that this dataset contains at least 1000 previously unannotated functional microproteins based on conservation of IEC smORFs against a variety of large and small mammals (Fig. 1c). Here, we present IEC_17915 and IEC_14429 as examples of microproteins with varying degrees of conservation. IEC_17915 is predicted by tBlastn to encode nearly identical protein sequence among all mammals tested (Fig. 1d), while IEC_14429 is only conserved between human and chimp indicative of its emergence and conservation in the primate lineage (Fig. 1e).

### Reproducibility and confidence in smORF calling

**Fig. 2:**
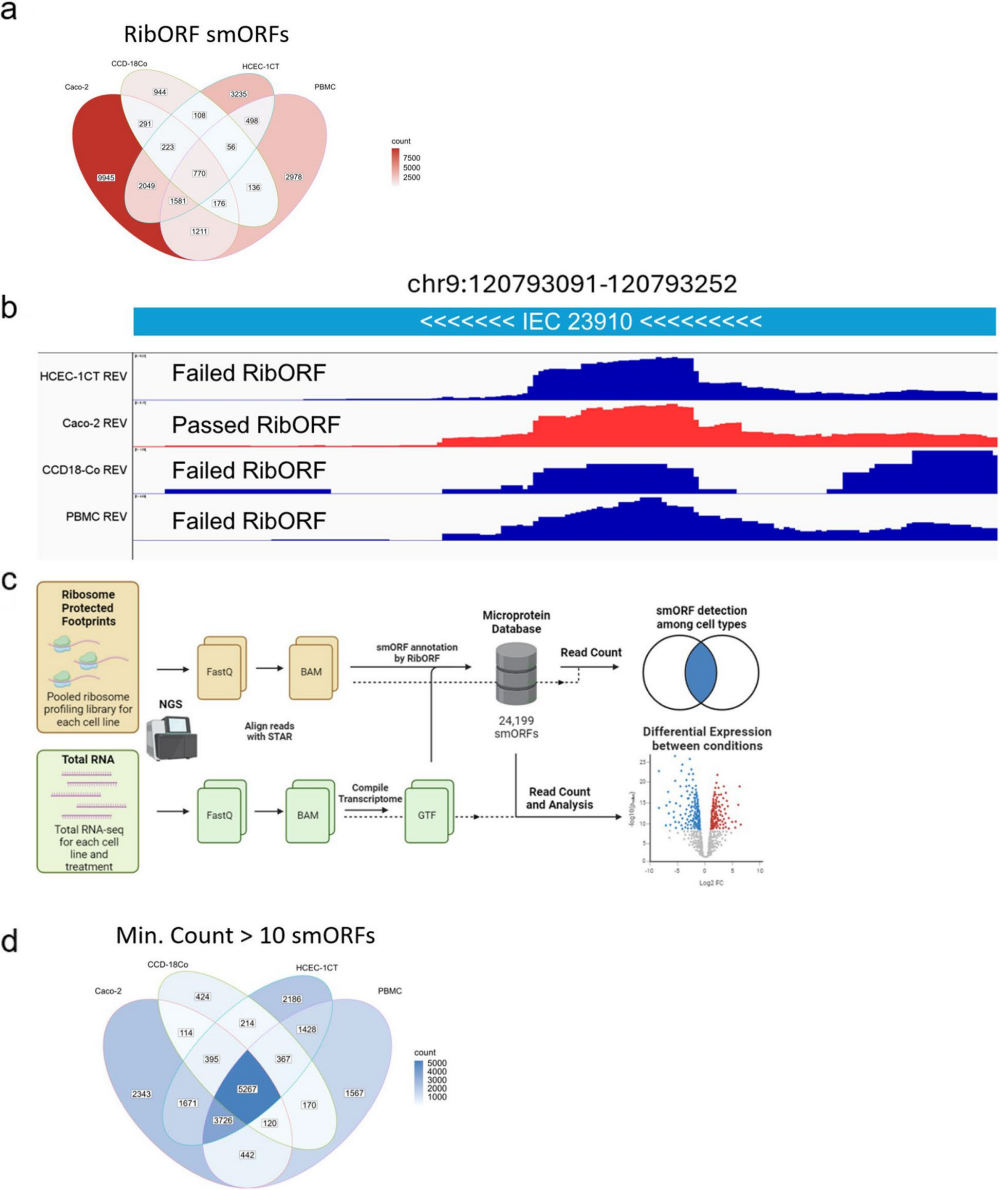
Distribution of smORFs among different cell lines. **a** Venn Diagram showing smORF called in various cell lines by RibORF only. Most smORFs (~ 70%) were predicted by RibORF only to be in their respective cell lines. **b** Example of ribosome profiling tracks on reverse strand (REV) of various cell lines, demonstrating the stringency of whether a smORF passing RibORF. In this case, only Caco-2 passed RibORF threshold (*P* > 0.8) while all other cell lines failed. **c** Diagram showing data analysis workflow. Both Ribosome Profiling and RNA-seq data were aligned by STAR to HG38. Using the RNA-seq data, full transcriptome of each cell line was compiled using STRINGTie. Using the Ribosome profiling data, the aligned reads were processed by RibORF with the predicted transcriptome to predict and annotate novel smORFs. The Ribosome profiling reads were then re-counted using HOMER to determine overall coverage among different cell types. Finally, the RNA-seq data were processed using HOMER and DESEQ2 for downstream analysis. **d** Venn Diagram showing smORF coverage among different cell line by read counts. Using a minimum read count threshold of 10 per cell line, only 27% of smORFs were found to be specific to each cell line

When we looked at the IEC smORF overlap among various cell lines we found that that 17,102 (or ~ 70%) IEC smORFs are only called in one cell line (Fig. 2a), suggesting that most translated smORFs are cell specific. However, it is important to note that our bioinformatic pipeline relies on RibORF to identify ORFs [46]. RibORF uses a score that combines (sm)ORF coverage and codon periodicity to score whether a (sm)ORF is being translated. Other (sm)ORF callers operate in a similar way and the final output depending on the acceptable score is a binary yes or no as to whether a (sm)ORF is being translated. In replicate studies, we observed that a smORF can be called in one replicate but not others, even though the ribosome coverage is excellent in all replicates or in different cell lines. For example, IEC_23910 is called as translated smORF in Caco-2 cells but not in HCEC, CCD18-Co, or PBMCs even though the ribosome coverage is excellent in all these cell lines (Fig. 2b), suggesting that the RibORF result is a false negative in these datasets. Similar observations have also been reported in the literature [47]. Because we see excellent reproducibility in ribosome coverage, we postulate that the false negatives must be due to differences in 3-nt periodicity between individual Ribo-seq replicates.

In theory, we could lower the RibORF scoring threshold to alleviate this false negative issue, but this would then lead to many false positives. Ideally, we’d like to know that a smORF call passed rigorous criteria in at least one dataset. To work around this, we modified our pipeline to include an additional step. Specifically, the RibORF output of translated smORF coordinates is fed into HOMER [51], and then we used HOMER to quantify the read counts in the RiboSeq data between the coordinates for every smORF that is called in at least one dataset. Thus, every smORF is initially assigned by RibORF but the translation of that smORF across samples is verified using ribosome coverage through HOMER (Fig. 2c).

Using a count threshold of ten in the RiboSeq data, we find that 17,679 (or ~70%) of IEC smORFs are found in more than one cell type (Fig. 2d), completely reversing the initial observation that only relied on RibORF alone. Lastly, if we applied an extremely relaxed count threshold of two in the RiboSeq data, 20,808 (or ~86%) IEC smORFs were found in more than one cell type, while 3492 (or ~14%) IEC smORFs remain unique to each cell type, suggesting that this analysis can preserve the specificity of smORFs among datasets (Fig. S1f). Thus, by adding an additional step to our pipeline we dramatically decrease false negatives in our calls and achieve greater confidence in the translation of the majority of the smORFs (Fig. 2d).

### Differential expression in RNA-seq datasets

Having built a robust dataset of smORFs from intestinal cells and PBMCs, we wanted to ask whether we could identify members of this family that are differentially regulated. For each of these conditions, we performed individual RNA-Seq experiments for each cell line and treatment condition to quantify changes in smORF mRNA expression under proinflammatory and profibrotic stimulation (Fig. 1a). We quantified differences in smORF expression using the established differential expression (DE) pipeline DESeq2 [52]. For example, we quantified the levels of all smORF-containing mRNAs to identify smORF mRNAs that are changing by TNFα treatment in CCD18-Co cells. This approach will miss microproteins that are regulated post-transcriptionally, but in past work we have found that the majority of changes in Ribo-Seq abundance is explained by changes in the underlying transcript amounts [20]. In total, sixteen experimental conditions were performed with 3 biological replicates each, for a total of 48 RNA-seq runs (Fig. 1a, Supplementary Table S3).

In order to confirm the efficacy of the different treatments among the cell lines, we first analyzed differential expression of known gene markers. For instance, under the conditions used for TNFα stimulation of Caco2 or HCEC-1CT cells we did not observe robust regulation of all inflammatory cytokines (Figure S2a, b). Optimization of the stimulation conditions and time points for particular cell lines could overcome some of these issues; however, we still had plenty of excellent datasets to test our hypothesis that we could identify smORFs that are regulated by inflammatory and fibrotic conditions, to highlight the use of DE in smORF research. Specifically, we used the following datasets for inflammation: Caco2 cells treated with interferon gamma (IFNγ), HCEC-1CT cells treated with IFNγ, CCD18-Co cells treated with TNFα and IL1β, and PMBCs with LPS and anti-CD3/CD28 antibodies. Further, CCD18-Co cells also demonstrated a robust response to the profibrotic stimulus TGFβ.

### smORFs regulated by IFNγ in Caco2 and HCEC cells

**Fig. 3:**
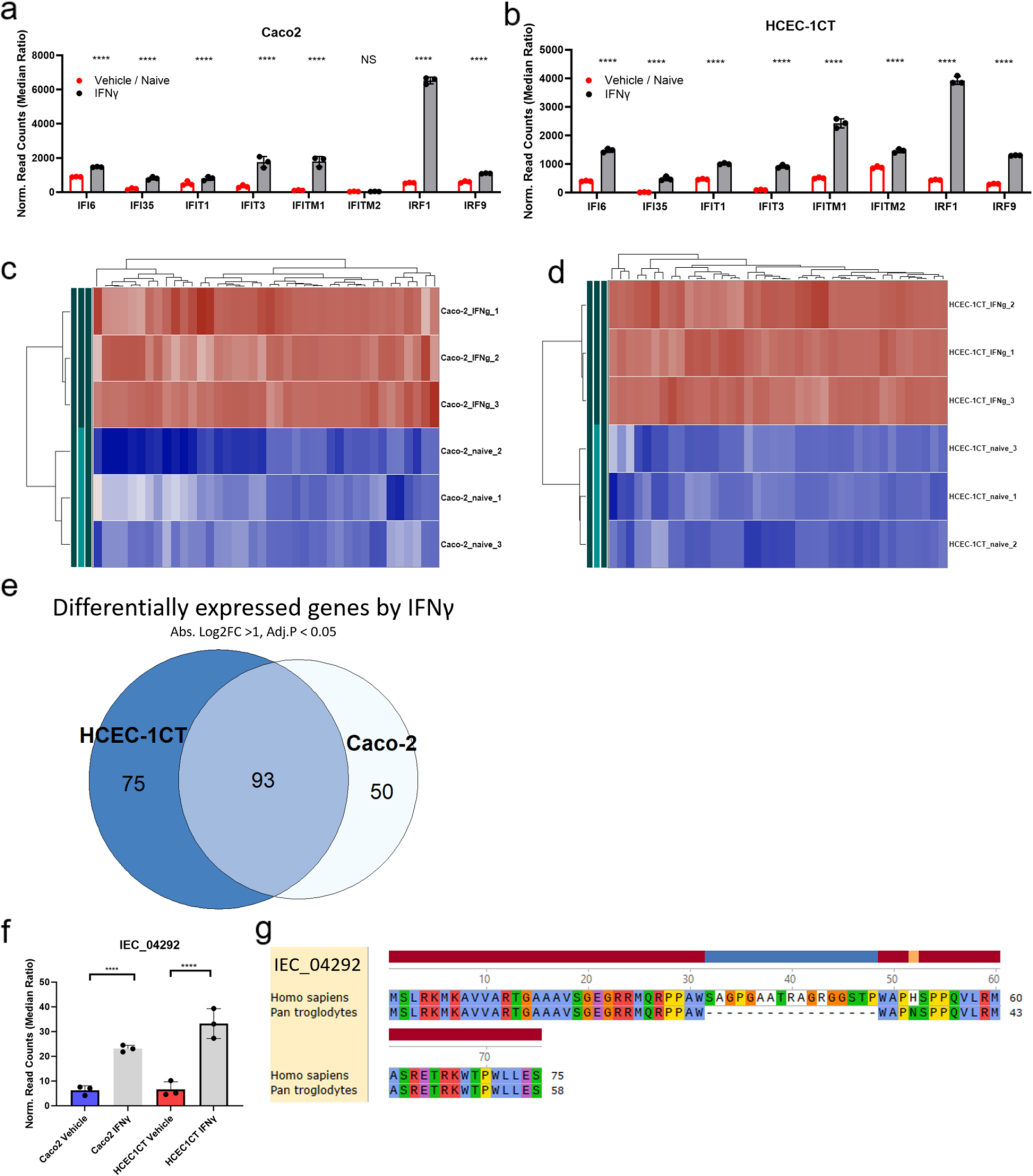
Effect of IFNγ on smORF expression in intestinal epithelial cells a, b) Gene expression of interferon inducible genes IFI6, IFI35, IFIT1, IFIT3, IFITM1, IFITM2, IRF1, IRF9 in Caco2 (**a**) and HCEC-1ct (**b**) cells treated with IFNγ. **c** Hierarchical heat map of top 40 smORFs differentially expressed smORFs in Caco2 cells. **d** Hierarchical heat map of top 40 smORFs differentially expressed smORFs in HCEC-1ct cells. **e** Venn diagram showing overlap of smORFs that are differentially expressed in HCEC-1ct or Caco2 cells (absolute Log2FC > 1, FDR < 0.05). **f**, **g** IEC_04292 overlaps the long non-coding RNA IRF1-AS1 and is increased upon IFNγ stimulation. Amino acid level conservation (**e**) of the predicted smORFs sequences to other species by tBLASTn. Bar graphs (a, b, f) showing normalized read counts. Differential expression analysis was performed through DESeq2, and comparisons were tested by Wald’s test followed by Benjamini and Hochberg correction, **** = FDR < 0.0001

IFNγ is a known stimulator of several cytokines, and we used the expression of canonical interferon-inducible genes (IFI6, IFI35, IFIT1, IFIT3, IFITM1, IFITM2, IRF1, and IRF9) to ensure that the experimental conditions used resulted in a predictable activation of these cells (Fig. 3a, b). Having established that known genes were changing as expected, we turned our attention to look at smORFs that are being regulated in these experiments. A heat map of up and down regulated smORFs reveals a robust signature induced by IFNγ treatment of Caco2 and HCEC-1CT cells (Fig. 3c and d), with large numbers of smORFs being upregulated upon IFNγ treatment.

To understand whether there is a conserved set of smORFs that are changed in these two cell lines that could be a part of a core IFNγ response, a Venn diagram was generated to examine the overlap. We found that of the 168 and 143 smORFs significantly differentially expressed in HCEC-1CT and Caco2 cells treated with IFNγ respectively, 93 were found to be differentially expressed in both cells (Fig. 3e). An analysis of the overlapping genes identified IEC_04292, a microprotein that is elevated up to 5-fold in IFNγ-treated cells (Fig. 3f). IEC_04292 is predicted to be encoded by the long non-coding RNA IRF1-AS1 and harbors significant regions of conservation between humans and chimps, as predicted by tBlastn (Fig. 3g).

### Regulation of IEC smORFs in CCD18-Co under inflammatory conditions

**Fig. 4:**
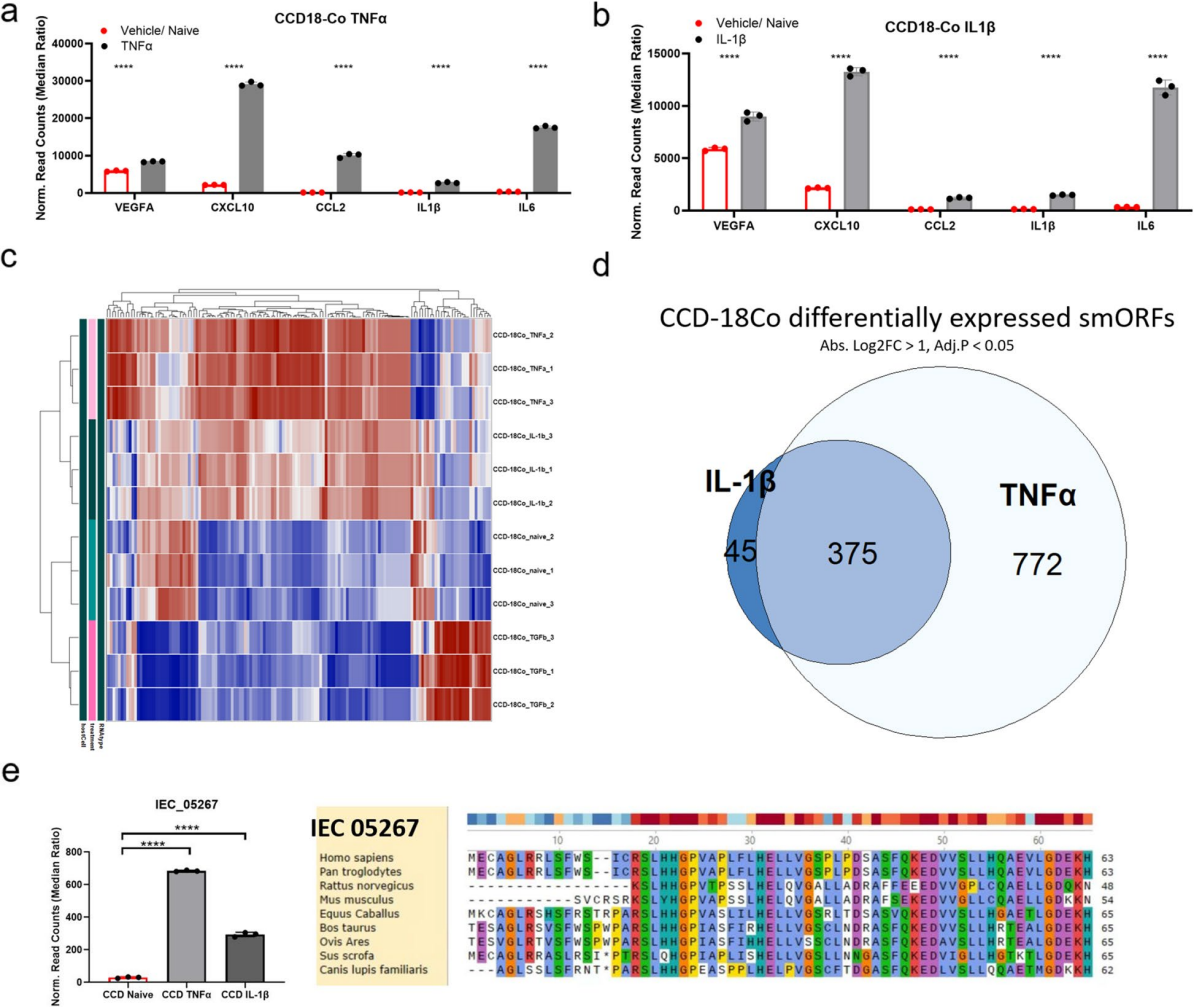
Differential expression of smORFs in CCD18-Co cells after stimulation with TNFα and IL-1β. a, b) Bar graph showing normalized read counts of inflammatory markers VEGFA, CCL2, CXCL10, IL1b, and IL6 in CCD-18Co cells treated with TNFα (**a**) and IL-1β (**b**) as compared to vehicle control (naïve cells). **c** Hierarchical heat maps of the top 40 differentially expressed smORFs in CCD-18co cells. **d** Venn diagram showing number of differentially expressed smORFs in CCD-18co cells (absolute Log2FC > 1, FDR < 0.05). Labels shown are specified treatments vs. control. **e **Upregulation of IEC_05267 in CCD cells upon treatment with TNFα and IL-1β. IEC_05267 is a highly conserved smORF that overlaps with an annotated non-coding RNA PSMB8-AS1 (chr6:32845552–32845740). Bar graphs (a, b, e) show normalized read counts of smORF. Differential expression analysis was performed through DESeq2, and comparisons were tested by Wald’s test followed by Benjamini and Hochberg correction, **** = FDR < 0.0001. Amino acid level conservation (e) of the predicted smORFs sequences to other species by tBLASTn

A look at marker gene (e.g., CXCL10, IL1β, IL6) expression in CCD18-Co cells treated with TNFα or IL1β demonstrated robust induction of these genes, validating these experimental conditions to strongly induce an inflammatory response in these cells (Fig. 4a and b). To test whether induction of different inflammatory stimuli in CCD-18Co cells control distinct IEC smORF expression changes or whether the signatures mostly overlap, we performed hierarchical heatmap analysis with the top 40 differentially expressed IEC smORFs (Fig. 4c). We observed overlapping IEC smORF expression patterns in TNFα- and IL-1β-treated cells (Fig. 4d), with most of the IL-1β-induced genes also regulated by TNFα. By contrast, many of the TNFα-regulated smORFs are specific to this condition. This can also be observed when we expand the heatmap dataset to include all differentially expressed smORFs (Fig S3a).

One of the shared smORFs is IEC_05267, an IEC smORF that overlaps with the long non-coding (lnc)RNA PSMB8-AS1. This smORF is significantly upregulated in CCD18-Co cells treated with either TNFα or IL-1β (Fig. 4e). Furthermore, tBLASTn reveals that the predicted protein sequence of IEC_05267 is conserved across various large mammals, but the N-terminus peptide is lost when aligning to dog and rodents (Fig 4e). Given that the lncRNA PSMB8-AS1 has previously been linked to the regulation of inflammation upon viral infection and cardiac inflammation [53, 54], our finding that this non-coding RNA encodes a microprotein suggests that this gene might also rely on this microprotein for its biology.

### CCD18 and TGFβ

CCD18-Co cells are a human colon fibroblast cell line and have been used previously as a model for intestinal fibrosis [43, 55]. Fibrostenosis is a hallmark of inflammatory diseases such as inflammatory bowel disease (IBD), and these experiments could identify smORFs involved in fibrostenotic biology. Furthermore, this experiment tests whether we can obtain unique pro-inflammatory (TNFα & IL-1β) and pro-fibrotic smORF signatures.

**Fig. 5:**
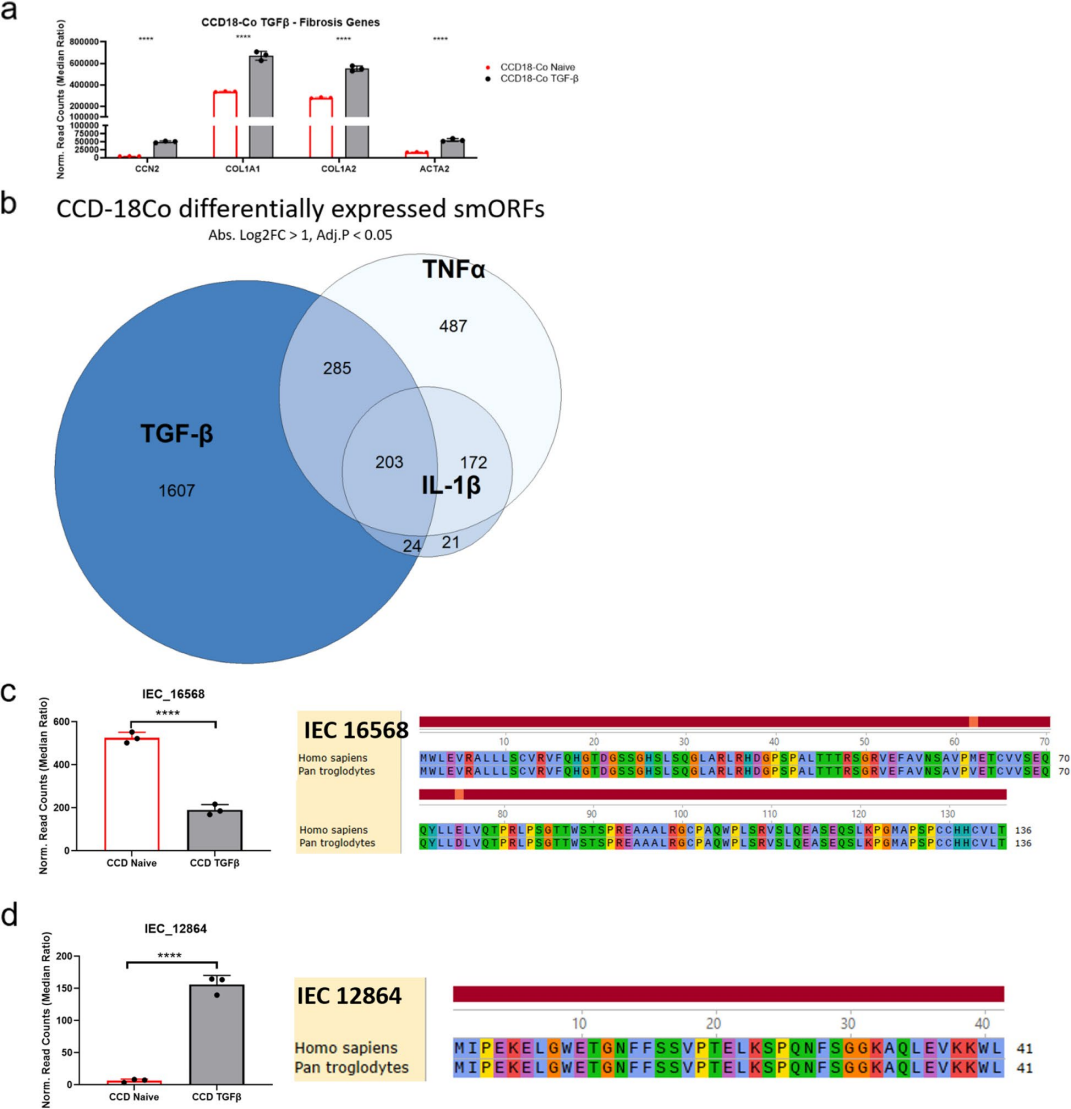
Differential expression of smORFs in CCD18-Co cells upon treating with profibrotic stimulant TGFβ. **a** Bar graph showing normalized read counts of fibrosis genes CCN2, COL1A1, COL1A2, and ACTA2 in CCD18-Co control cells vs. TGFβ treated cells. **b** Venn diagram showing number of differentially expressed IEC smORFs (absolute Log2FC > 1, FDR < 0.05) in CCD18-Co cells. Labels shown are specified treatments vs. control. **c** Downregulation of IEC_16568 in CCD18-Co cells treated with TGFβ to induce fibrosis. IEC 16,568 is a dORF of C1orf198 (chr1:230838598–230839005). **d** Upregulation of IEC_12864 in CCD18-Co cells treated with TGFβ to induce fibrosis. IEC 12,864 overlaps the predicted non-coding RNA LINC00545 (chr13:30869083–30869205). Bar graphs (a, c, d) showing normalized read counts of smORF. Differential expression analysis was performed through DESeq2, and comparisons were tested by Wald’s test followed by Benjamini and Hochberg correction, **** = FDR < 0.0001. Amino acid level conservation (c, d) of the predicted smORFs sequences to other species by tBLASTn

Treatment of CCD18-Co cells with TGFβ induced pro-fibrotic genes CCN2, COL1A1, COL1A2, and ATCA2 (Fig. 5a). Comparison of the hierarchical heatmap analysis with the top 40 differentially expressed IEC smORFs revealed that CCD18-Co cells treated with TNFα and IL-1β are mutually exclusive from control and TGFβ-treated cells (Fig. 4c). Furthermore, we observed distinct IEC smORF expression patterns between control, TNFα & IL-1β, and TGFβ-treated cells. A Venn diagram of the different conditions shows that most of the TGFβ-regulated smORFs are unique to TGFβ (Fig. 5b).

We looked for conserved smORFs regulated by TGFβ and found IEC_16568 and IEC_12864. IEC_16586 overlaps the 3’UTR region of an annotated gene, C1ORF98, and is significantly downregulated upon activation of fibrosis in CCD18-Co cells by TGFβ and is otherwise minimally changed under inflammatory stimuli (Fig 5c). Additionally, tBLASTn predicts protein coding sequence of high similarity within the chimp genome for IEC_16568 (Fig 5c). Next, IEC_12864 is a smORF found to be encoded by the lncRNA LINC00545 and is significantly upregulated upon activation of fibrosis by TGFβ in CCD18-Co cells (Fig. 5d). IEC_12864 is predicted by tBLASTn in the chimp genome to contain coding sequence of high similarity (Fig 5d). Finally, we would like to highlight IEC_23988, which is a smORF that is found to be overlapping with the predicted novel protein ENSG00000222032. IEC_23988 is a smORF that is significantly upregulated in CCD18-Co cells treated with TGFβ and may play a role in fibrosis of intestinal epithelial cells (Fig. S3b) However, tBLASTn searches across multiple species reveal poor conservation of IEC_23988 to other large mammals, all missing the N-terminal translational start codons (Fig S3b). Taken together, these data reveal IEC smORF regulation among different profibrotic stimulatory pathways and provide a pool of smORFs for downstream functional validation studies.

### Regulation of IEC smORFs in PBMCs

**Fig. 6:**
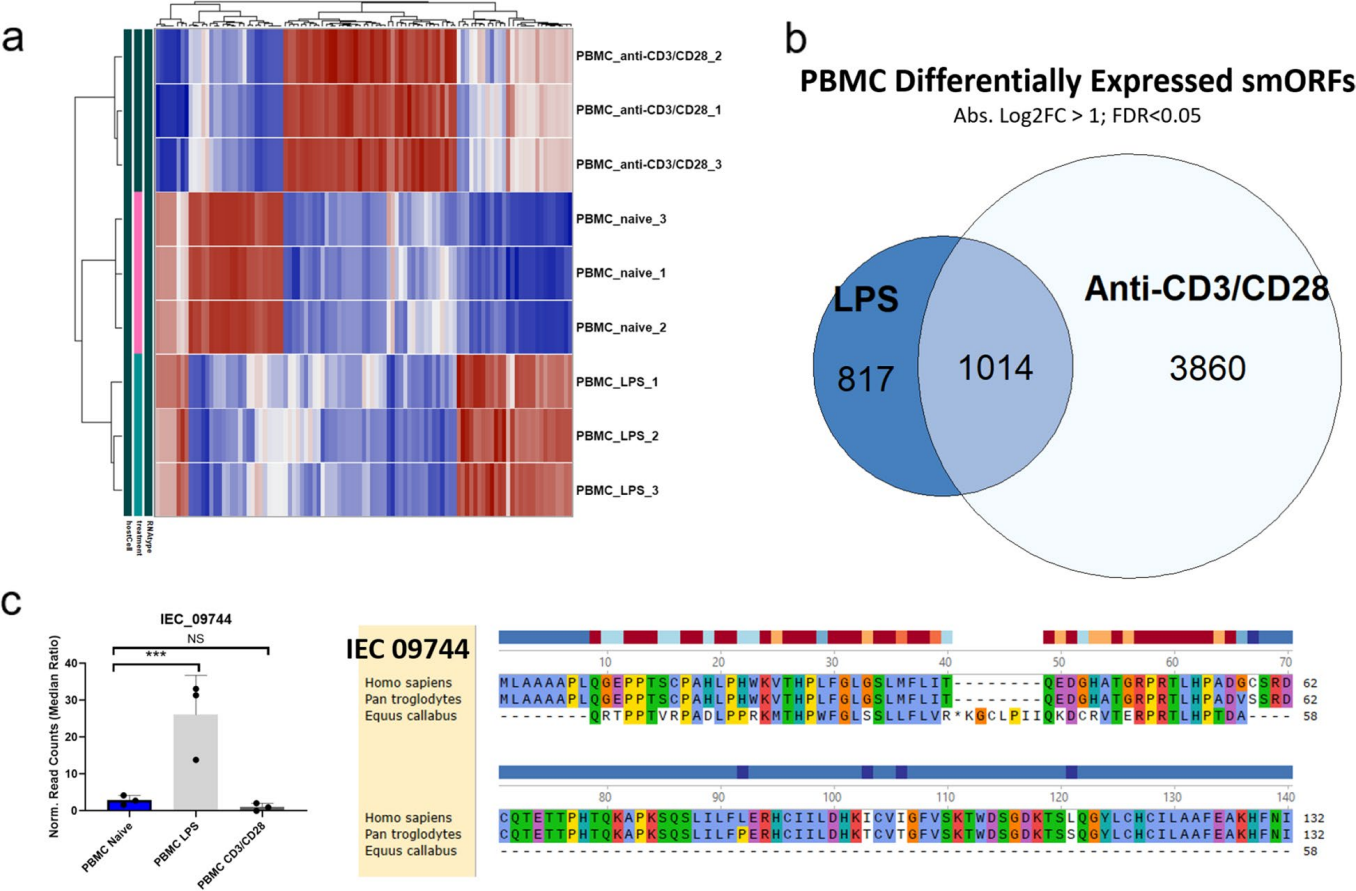
Differential expression of smORFs in PBMCs after treatment with LPS to induce inflammation and anti-CD3/CD28 to activate T-cells. **a** Hierarchical heat maps of the top 40 differentially expressed smORFs in PBMCs treated with LPS, anti-CD3/CD28, and vehicle control (naïve). **b** Venn diagram showing overlap of differentially expressed genes in PBMCs. Labels shown are specified treatments vs. control. **c** Upregulation of IEC_09744 in PBMCs treated with LPS, but not cells treated with anti-CD3/CD28. IEC_09744 overlaps with an annotated non-coding RNA LINC00239 (chr14:101731806–101732460). Bar graphs (**c**, left) show normalized read counts of smORF. Differential expression analysis was performed through DESeq2, and comparisons were tested by Wald’s test followed by Benjamini and Hochberg correction, **** = FDR < 0.0001. Amino acid level conservation (c, right) of the predicted smORFs sequences to other species by tBLASTn

Next, we looked at PBMCs, which are not part of the intestine, but can migrate to all tissues during inflammatory processes and are therefore relevant in mediating the fibrotic phenotype. Treatment of PBMCs with the inflammatory stimuli lipopolysaccharide (LPS) or T-cell activating antibodies anti-CD3/CD28 significantly increases inflammatory markers, including CCL2, IL-1β and IL6 (Fig. S4a, b). Furthermore, anti-CD3/CD28 treatment in PBMCs significantly increases T-cell activation as measured by gene expression of IL2 and IL2RA (CD25) (Fig. S4c). To examine whether IEC smORF expression is regulated by different stimulants in PBMCs, we performed hierarchal heatmap analysis of the top 40 (by fold change) significant IEC smORFs. This analysis revealed distinct clusters of significantly differentially expressed smORFs in each condition (Fig. 6a). For example, we observe a large cluster of IEC smORFs that are significantly upregulated upon T-cell activation by anti-CD3/CD28 in PBMCs. A smaller cluster of IEC smORFs can be found where there is significant down regulation in both LPS and anti-CD3/CD28 treated PBMCs. In total, there are 1014 smORFs that are differentially expressed across both conditions, and 817 and 3860 smORFs that are uniquely differentially expressed in either LPS or anti-CD3/CD28 treated PBMCs, respectively (Fig. 6b). Interestingly, these clusters are less pronounced in the hierarchical heatmap for all differentially expressed smORFs, suggesting that a significant number of smORFs identified are either not well differentially expressed among different PBMCs or not highly expressed at all (Fig. S4d).

One highlighted example of a smORF regulated in PBMCs is IEC_09744. This smORF overlaps the annotated non-coding RNA LINC00239 and is significantly upregulated upon LPS treatment (Fig. 6c). IEC_09744 is predicted by tBLASTn to be conserved across the whole protein sequence between human and chimps and has a conserved 31 amino acid domain that is missing the translational start site in horses (Fig. 6c). Another example is IEC_10748, which is a smORF encoded on the 5’ end of the pseudogene LGALS17A. Although not well conserved, this smORF is induced by both LPS and anti-CD3/CD28 in PBMCs, as well as IFNγ-treated HCEC-1CT cells (Fig S5a, S5b). Thus, one possible hypothesis is that IEC_10748 is involved in intestinal inflammatory response through the TLR4-IFNγ pathway.

Other highlighted examples include two IEC smORFs that are hypothesized to be involved in T-cell activation. Both IEC_2033 and IEC_05820 are predicted IEC smORFs that overlap with annotated non-coding RNAs (LINC00861 and LINC00649, respectively) and demonstrate significant downregulation upon treatment with anti-CD3/CD28 (Fig. S5c, S5e). Furthermore, both IEC smORFs are predicted to be encoded also in the pan troglodyte genome (Fig. S5d, S5f). Altogether, these results demonstrate that we can detect IEC smORF regulation in PBMCs upon TLR4 activation by LPS or T-cell activation by anti-CD3/CD28 treatments.

### Regulation of smORFs in inflammatory bowel disease

**Fig. 7:**
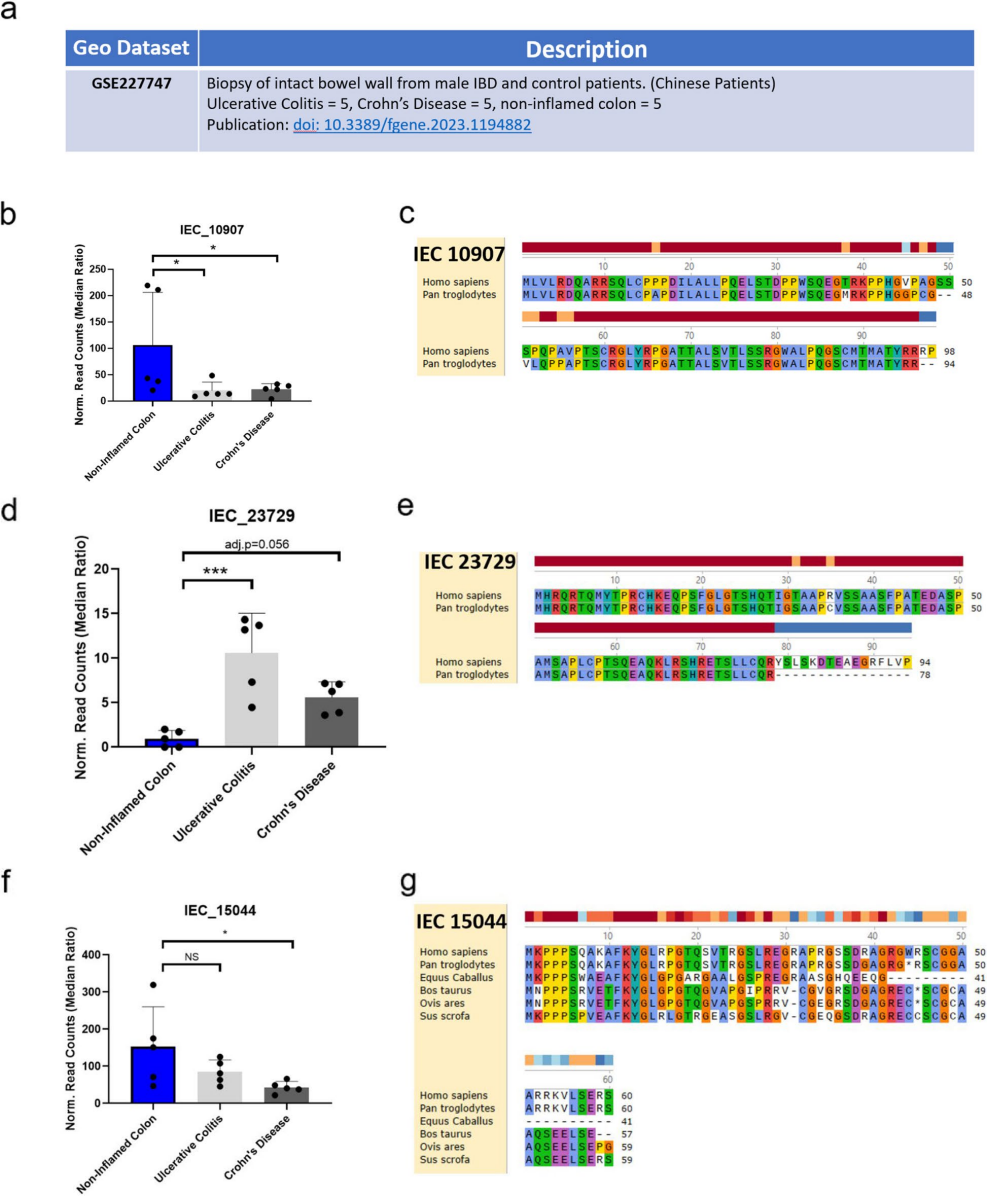
Differential expression of smORFs in human inflammatory bowl disease biopsies. **a** RNA-seq data used in this analysis were previously deposited in NIH GEO datasets, GSE227747. **b**-**g**) Examples of smORFs differentially expressed among both UC and CD patients (**b**, **c**) UC patient only (**d**, **e**) and CD patients only (**f**, **g**). IEC_10907 is found on the annotated non-coding RNA SLC9A3-AS1 (chr5:476370–477812). IEC_23729 is found on the annotated non-coding RNA LINC01484 (chr5:173709124–173719073). IEC_10544 is found on the intron of OAZ1 (chr19:2270380–2270427). Bar graphs (**b**, **d**, **f**) shown are normalized read counts among RNA-seq samples. Differential expression analysis was performed through DESeq2, and comparisons were tested by Wald’s test followed by Benjamini and Hochberg correction, * = FDR < 0.05, *** = FDR < 0.001. Amino acid level conservation of predicted smORFs are shown in (**c**, **e**, **g**)

To identify smORFs that are regulated by inflammatory bowel (IBD), we analyzed publicly available RNA-Seq samples of colon epithelial cells from adult IBD patients (GSE227747) and pediatric IBD patients (GSE117993) (Fig. 7a). The adult patient cohort consists of biopsy samples from Crohn’s disease (*n* = 5), Ulcerative colitis (*n* = 5), and non-inflammatory control patients (*n* = 5). Differential expression analysis identified 358 IEC smORFs with FDR < 0.05 (Fig. S6a, b), which is significantly fewer than the ~ 1,000 differentially expressed smORFs found in our cell line data (Figs. 4d, 5b and 6b). This difference is likely due to the greater variability between human samples. Generally, PCA of human patient data tends to show poorer clustering compared to in vitro cell line data, and we observe a similar pattern here (Fig. S6c). The high variance between human samples limits the number of significant smORFs detected, particularly with a small sample size. Nevertheless, of the 358 differentially expressed IEC smORFs, 31 were predicted by tBLASTn to have highly similar sequences in the mouse or rat genome (Fig. S6d, Supplementary Table S2), providing a pool of candidates for further validation in IBD rodent models. Next, we generated hierarchical heatmaps on the top 40 (by fold change, FDR < 0.05) differentially expressed smORFs and clustering of IBD samples was observed (Fig. S6e). Here, we would like to highlight three example IEC smORFs whose gene expression correlates with disease condition. First is IEC_10907, which is significantly downregulated in both UC and CD patient biopsy samples (Fig. 7b, c). IEC_10907 overlaps with the non-coding RNA SLC9A3-AS1 and is predicted to be conserved between humans and chimps (Fig. 7c). Another similar example is IEC_23729, which is significantly upregulated in UC biopsies compared to control tissue (Fig. 7d). IEC_23729 has a similar trend in upregulation in CD patients but is not significant (FDR = 0.056). IEC_23729 overlaps on the non-coding RNA LINC01484 and its predicted amino acid sequence is well conserved between human and chimps (Fig. 7e). Finally, IEC_15044 is an IEC smORF that is downregulated only in CD patients (Fig. 7f). This smORF is found on the intron of OAZ1 and its amino acid sequence can be found in genomes of multiple large mammalian species (Fig. 7g). Taken together, these examples represent top candidates for downstream functional validation studies.

### Validation of IEC smORFs by mass spectrometry

**Fig. 8:**
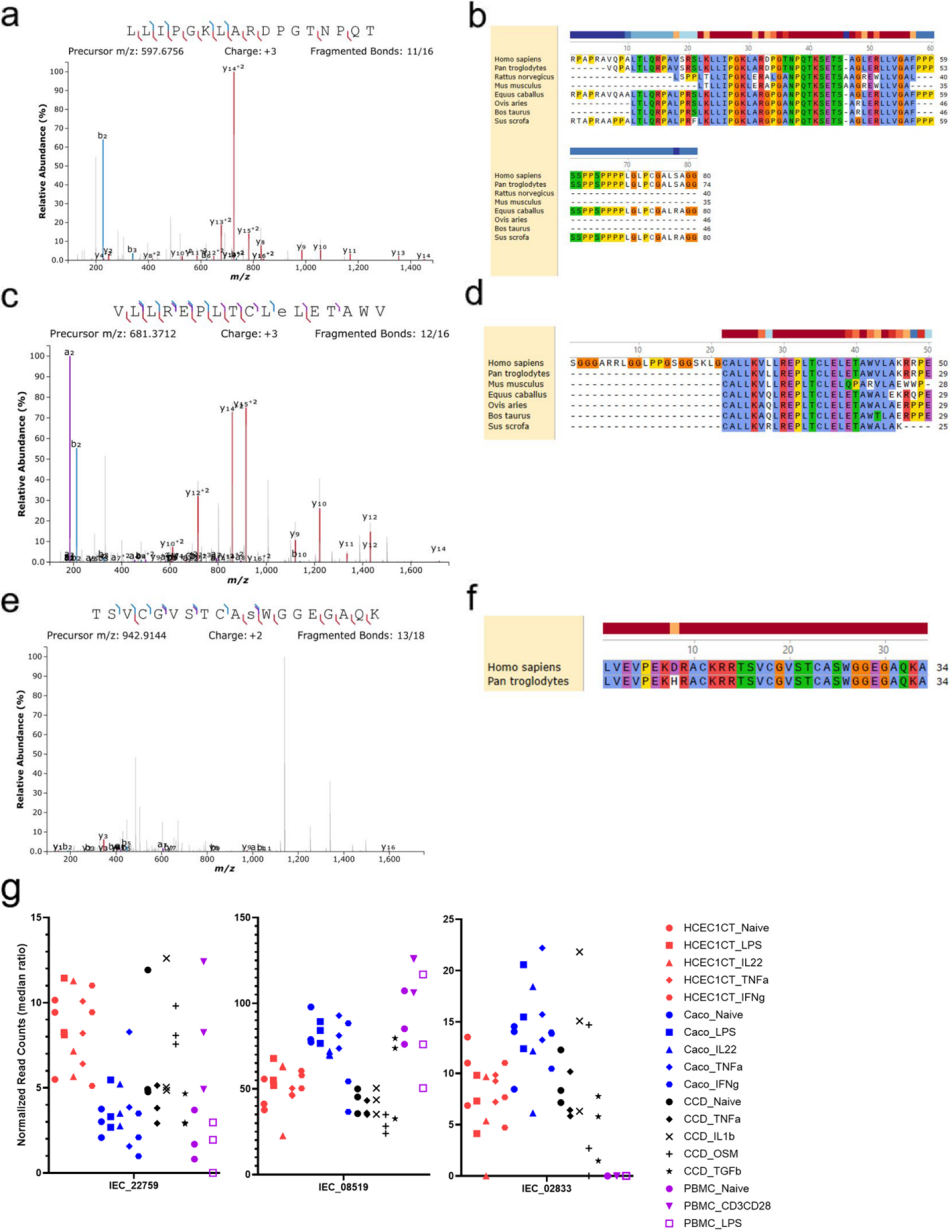
Identification of smORFs by mass spectrometry in Caco2 cells. **a**-**b**). Representative MS spectra of IEC_22759 (**a**) and amino acid level conservation of predicted sequences by tBLASTn (**b**). IEC_22759 overlaps the non-coding RNA LPP-AS2 (chr3:188153817–188154056) c-d). Representative MS spectra of IEC_08519 (**c**) and amino acid level conservation of predicted sequences by tBLASTn (**d**). IEC_08519 overlaps the 5’UTR of CHCD7 (chr8:56211688–56211837). e-f). Representative MS spectra of IEC_02833 (**e**) and amino acid level conservation of predicted sequences by tBLASTn (**f**). IEC_02833 overlaps the 5’UTR of GPRC5C (chr17:74431666–74431767). **g**) Dot plot showing normalized read counts of MS detected smORFs in RNA-seq samples. Differential expression analysis by DESeq2 reveals no significant change among samples

We used mass spectrometry to provide direct evidence of microproteins from the smORFs. First, we generated our own proteomic dataset from Caco2 cells using previously established protocols to enrich small proteins [16]. From these experiments, we found unique peptides for 27 microproteins (Supplementary Table S4). Representative spectra demonstrate good fragment ion coverage. IEC_22759 (Fig. 8a) overlaps the non-coding RNA LPP-AS2 and is well conserved across multiple species (Fig. 8b). Similarly, IEC_08519 (Fig. 8c) is found on the 5’UTR of CHCD7, though only its C-terminal peptide is well conserved across different species (Fig. 8d). Finally, IEC_02833 (Fig. 8e) is found on the 5’UTR of GPRC5C and its predicted amino acid sequence is only found in the chimp genome (Fig. 8f). Based on the RNA-seq analysis, both IEC_22759 and IEC_08519 are ubiquitously expressed and not induced by any stimulant in all three cell lines (Fig. 8g). However, IEC_02833 is found to be intestinal cell specific as its expression cannot be detected in PBMC cells (Fig. 8g), suggesting an intestinal cell-specific function. Next, we re-analyzed published HLA-I proteomic datasets (PXD013649), as immunoprecipitation of HLA-I enriches small peptides and enhances microprotein detection. Of the 24,199 smORFs, we found unique peptides from 70 microproteins (Supplementary Table S5). Differential analysis and subsequent hierarchical heatmap of our RNA-seq data show that half of these IEC smORFs are highly expressed in all RNA seq samples, while a few, such as IEC_18903 and IEC_12988 are found only in CCD18-Co cells (Figure S7a). Taken together, these results demonstrate evidence for specific microproteins enriched in intestinal epithelial cells.

## Discussion

The overarching goal of this work was to determine if we could identify microproteins with potential roles in tissue homeostasis and disease by integrating our microprotein discovery pipeline (Fig. 1a) with differential expression data. The data source can vary and we demonstrate the use of RNA-Seq data we collected from differentially stimulated intestinal cell lines or the use of publicly available clinical data.

These efforts began by collecting Ribo-Seq data from intestinal cells and PBMCs treated with a variety of pro-inflammatory and pro-fibrotic stimulants. We’ve previously experienced low overlap between Ribo-Seq datasets [20]. An analysis of the Ribo-Seq coverage indicated that the ORF caller is stringent and similar ribosome coverage do not always pass the rigorous filters of these callers [46] (Fig. 2b). To provide more confidence in the calls, we provide a simple solution that looks at smORF coverage across multiple datasets for an smORF that is called in at least one dataset. This solution suggests that at least 70% of our calls overlap giving us higher confidence in our predictions. Of course, for any smORF that becomes the subject of biological studies we highly recommend visual curation of the primary Ribo-Seq data.

In this study, we highlight smORFs that demonstrate varying degrees of conservation. While there is substantial evidence supporting the functionality of smORFs that are not highly conserved [34, 35, 56], we propose that conserved microproteins remains the strongest candidates for downstream validation studies. For example, our smORF database contains novel microproteins that are highly conserved, such as IEC_17915 (Fig. 1d), from human-to-mouse. Previous examples of microproteins with this level of conservation have proven to be functional [57, 58], and we fully anticipate this microprotein to also have a disease-mediating function. Moreover, we find many other microproteins, for example IEC_14429, which are nearly identical between humans and chimps. Unlike the more highly conserved microproteins, we needed additional data to pursue the study of primate-specific microproteins. While non-conserved microproteins with significant differential expressions are not highlighted within the main figures, they can be explored within the supplementary tables and may be of interest to future studies in the field.

Consequently, we explored whether biological regulation would serve as a suitable approach to obtain additional data to focus on specific microproteins. Indeed, treatment of intestinal cell lines with different pro-inflammatory stimuli identified numerous interesting microproteins. For example, IEC_04292 is significantly upregulated in Caco2 and HCED-1CT cells treated with IFNγ (Fig. 3) and stimulation of CCD-18Co cells with TNFα or IL-1β led to increased expression of an RNA PSMB8-AS1 (Fig. 4e). PSMB8-AS1 is annotated as a non-coding gene, but within it our ribosome profiling identified IEC_05267 that produces a 63-amino acid microprotein with conservation at the amino acid level with chimps, horse, and pig (examples with a methionine start codon) but no evidence of a translated smORF in mice and rats (Fig. 4e).

The non-coding RNA PSMB8-AS1 has been linked to biology and disease. In one study, it was discovered that PSMB8-AS1 is increased in human atherosclerotic plaques, and generation of a PSMB8-AS1 knockin mouse showed that this mRNA is sufficient to drive disease in an atherosclerotic mouse model [53]. Additionally, there are several studies in pancreatic cancer [59] and glioma [60, 61] that demonstrate a link between PSMB8-AS1 and neoplastic growth and maintenance. Finally, PSMB8-AS1 has also been linked to autoimmune disease (scleroderma and lupus) [62, 63] and the control of viral infection, providing additional support for our observations that this mRNA is upregulated under inflammatory conditions. Based on these results, it is enticing to hypothesize that the microprotein might be responsible for some of the biological functions and roles in disease that have been attributed to PSMB8-AS1 non-coding RNA. This example highlights the value of these efforts as we are able to identify microproteins and, because of their regulation or roles of their host RNAs, develop a hypothesis about their contributions to mediating a disease phenotype.

These examples highlight the ability of gene expression to identify microproteins with potential biological functions in cases where conservation is limited to the primate lineage. To ensure that these microproteins are not specific to these cells or related to cellular stress with pro-inflammatory stimuli, we also looked at the impact of pro-inflammatory stimuli (LPS, anti-CD3/CD28) on PBMCs and pro-fibrotic stimulus TGFβ on CCD18-Co intestinal fibroblasts.

In PBMC’s, we have identified several annotated long non-coding RNAs from our ribosome profiling dataset. One caveat in our PBMC analysis is that this heterogenous cell population having varying effects upon stimulation, and differential expression analysis in PBMCs in-bulk is not as effective as using flow cytometry or single-cell methods to differentiate minute differences among cell populations. Fortunately, we can use published single-cell expression studies on PBMCs to hypothesize the effect of LPS and anti-CD3/CD28 on specific PBMC cell types [64]. For example, our dataset predicted two protein-coding lncRNAs LINC00239 and LINC00861. Both lncRNAs have been previously implicated in various cancer models. First, overexpression of LINC00239 is correlated with poor colorectal cancer and esophageal squamous cell carcinoma prognoses [65–67]. Decreasing LINC00239 expression using targeted siRNAs inhibited cell proliferation and migration of various cancer cell types [65–67]. From our ribosome profiling experiments, LINC00239 is predicted to encode IEC_09744, a 132 amino acid microprotein with conservation between human and chimps (Fig. 6c). Here, we find IEC_09744 to be upregulated upon stimulation with LPS, but not anti-CD3/CD28 in PBMCs, suggesting an innate pro-inflammatory role to the microprotein. Furthermore, since single-cell expression studies have shown that LPS stimulation in PBMCs mainly results in monocyte activation with no significant changes in cell population [64], we can hypothesize that IEC_09744 activation is mainly driven through monocyte activation as a response to LPS stimulation. Similarly, LINC00861 has been shown to be important in the immune response in lung adenocarcinoma, where higher levels of the non-coding RNA correlate with improved cancer prognosis [68]. Our data shows that LINC00861 encodes a 35-amino acid microprotein, IEC_20333, which is significantly downregulated upon T-cell activation by anti-CD3/CD28 (Fig. S5c, d). Given that single-cell expression analysis of PBMCs has shown anti-CD3/CD28 stimulation leads to significant monocyte depletion and robust activation of both CD4 + and CD8 + T cells [64], this suggests two possibilities: either IEC_20333 is primarily expressed in monocytes and is depleted as monocytes are lost during T-cell activation, or it is downregulated in activated T cells and may play a role in the maintenance of naïve T cells. Taken together, we hypothesize that microproteins can be encoded from non-coding RNAs that play roles in innate immunity, tumor neogenesis and maintenance. Future work on PBMCs should use single-cell methods such as fluorescence activated cell sorting to elucidate differential smORF regulation among different immune cell populations to test specific hypotheses.

In CCD18-Co cells we observe a set of smORF mRNAs that are regulated by TGFβ when compared to the pro-inflammatory stimuli, revealing that microprotein expression is pathway specific. Indeed, this experiment reveals two primate-specific microproteins that are downregulated (IEC_16568) and upregulated (IEC_12864) by TGFβ indicating a potentially functional role in TGFβ-mediated fibrosis.

We then asked whether clinical RNA-Seq data could be used to identify disease-associated microproteins. We selected adult ulcerative colitis and Crohn’s disease patients from publicly available RNA-seq data from intestinal biopsies to perform differential expression analysis on these smORFs. Once again, this analysis found several microproteins that are up- or down-regulated in ulcerative colitis or Crohn’s disease; including the strongly-upregulated IEC_23729 which appears to be triggered by these intestinal inflammatory diseases. Although these in vitro cell lines represent divergent cell types within the colon, these results allude to the cell specificity of smORF expression within the inflamed colon epithelium.

## Conclusion

In summary, this study demonstrates the value of using differential expression analysis to identify microproteins that may lack extensive conservation but still hold biological significance. As shown by the example of Aw111020, it is possible to uncover non-conserved microproteins with important roles in physiology and inflammation [ref]. The microproteome remains a largely untapped resource for discovering such genes, and our efforts here demonstrate the potential to identify disease-regulated microproteins for further investigation.

Notably, if we relied solely on conservation, only around 6% of smORFs would appear functional based on their conservation between humans and mice. This is likely an underestimation and many more functional microproteins may exist. In this study, we also highlight several microproteins that are conserved only between humans and chimpanzees, which would often be not included when creating a shortlist of microproteins for follow-up studies without incorporating differential expression analysis.

We hope that these data can provide a foundation for more sophisticated informatic studies and encourage others to revisit their RNA-Seq datasets to explore whether any of the microproteins we have annotated could play a role in intestinal homeostasis or disease. Given that none of these microproteins have been previously studied, they serve as a potential new set of biomarkers that, with further research, may be shown to play causal roles in disease biology.

## Detailed methods

### Preparation of various cell lines for ribosome profiling and RNA-sequencing

For preparation of cells for RNA sequencing, cells were seeded into 6 well plates according to the following densities: CCD18-Co cells (ATCC, Cat# CRL-1459) 0.47 × 10^6^ cells/1.5mL of EMEM + 10% FBS/well (x 2 wells), CaCo2 cells (ATCC, Cat# HTB-37) 0.51 × 10^6^ cells/1.5mL of EMEM + 10% FBS/well (x 2 wells), HCEC-1CT cells (ABM, Cat# T0715) 1.5 × 10^6^ cells/1.7mL of DMEM + 2%FBS, 20ng/mL hEGF (Sigma-Aldrich, Cat# E9644), 10ug/mL Insulin (Sigma-Aldrich, Cat# I9278), 2ug/mL Apo-Transferrin (Sigma-Aldrich, Cat# T2036), 5nM Sodium-Selenite (Sigma-Aldrich, Cat# S5261), 1ug/mL Hydrocortisone (Sigma-Aldrich, Cat# H0396), PBMC (Hemacare, Cat# PB009C-3, lot:20063132) (4 × 10^6^ cells/4 mL RPMI + 2% human serum) (x 2 wells). For Ribosome Profiling studies cells were seeded into larger format plates according to the following densities: CCD18-Co cells 3 × 10^6^ cells/23mL in 15 cm plate, CaCo2 cells 4.2 × 10^6^ cells/10mL in 10 cm plate, HCEC-1CT cells 5 × 10^6^ cells/8.7mL in 10 cm plate, PBMC 39 × 10^6^ cells/39mL in T75 flask. Cells were then treated with following stimulants (all 1uL/mL of media) for 24 h before being harvested: LPS (100ng/mL) (Sigma-Aldrich, Cat# L4391-1MG), IL-22 (10ng/mL) (RnD Bio, Cat# 782-IL), TNFa (10ng/mL) (RnD Bio, Cat#210-TA-005), IFN-g (10ng/mL) (RnD Bio, Cat#285-IF-100), IL-1b (10ng/mL) (RnD Bio, Cat#201-LB010), OSM (10ng/mL) (RnD Bio, Cat# 295-OM-010) and TGFb (10ng/mL) (RnD Bio, Cat#240-B-010). After 24 h, for RNAseq samples, cells were washed with PBS followed by addition of 500uL TRIZOL to each well. Two wells of each sample were pooled and stored at -80 °C until sequencing. For RIBOseq samples, cells were washed twice after treatment with ice cold PBS + 100ug/mL cycloheximide (CHX; Fisher Scientific, AAJ66004X) and snap frozen in liquid nitrogen and stored at -80 °C.

### Ribosome profiling library preparation and sequencing

Library preparation for Ribosome Profiling was carried out as previously described [20]. Briefly, cells were washed twice after treatment with ice cold PBS supplemented with 100ug/mL cycloheximide (CHX; Fisher Scientific, AAJ66004X) and snap frozen in liquid nitrogen and stored at -80^o^C. After thawing briefly on ice, the cells were lysed with 300uL of ice-cold lysis buffer (20mM Tris-HCl, pH 7.4, 150mM NaCl, 5mM MgCl_2_, 1% Triton X-100, 1mM DTT, 25U Turbo DNase (Thermofisher Cat# AM2238), and 100ug/mL CHX – added fresh) for 10 min with periodic vortexing and pipetting through an 18G needle to ensure the cells are dispersed. Cell lysates were clarified by centrifugation for 15,000 g for 10 min at 4^o^C, flash frozen in liquid nitrogen, and stored in -80^o^C for up to 1 week. For each Abm HCEC-1CT, Caco2, CCD118co, and PBMC pool of unstimulated and stimulated cells, ribosome footprinting was carried out by digesting 50 µg of RNA in 300 µL lysate with 0.375 U/µg RNase I (Lucigen, N6901K) for 45 min at room temperature. Digestion reactions were quenched with 200 U Superase-In RNase I inhibitor (Thermo Fisher, AM2694) on ice. Following digestion, monosomes were purified from small RNA fragments using Micro-Spin S-400 h columns (GE Life Sciences), and ribosome protected RNA fragments (RPFs) were extracted by acid phenol chloroform and isopropanol precipitation. Sequencing libraries were prepared as in McGlincy and Ingolia with some modifications [69]. First, the RiboZero Mammalian Kit (Illumina) was used to deplete rRNA after RPF extraction and just prior to RPF size selection by gel extraction. Second, the Zymo clean & concentrator step after adaptor ligation is omitted and the reaction was carried over straight into reverse transcription. For the reverse transcription step to form cDNA, Episcript RT (Lucigen, ERT12910K) was used. Following reverse transcription, excess primer was degraded using Exonuclease I (Lucigen, X40520K) and the RNA templates were degraded using Hybridase (Lucigen, H39500). For the cDNA circularization step, CircLigase I (Lucigen, CL4111K) was used. PCR amplification was then carried out using Phusion Hot Start II High-Fidelity Master Mix (Thermo Fisher, F565L) for 9–12 cycles.

The adapters and primers for library construction used were as follows:

3’ adapter – 5’ /5phos/AGATCGGAAGAGCACACGTCTGAA/3ddC/-3’;

RT primer – 5’ /5Phos/AGATCGGAAGAGCGTCGTGTAGGGAAAGAG/iSp18/GTGACTGGAGTTCAGACGTGTGC C-3’;

PCR forward primer – 5’-AATGATACGGCGACCACCGAGATCTACACTCTTTCCCTACACGACGCTC-3’ ;

Illumina TruSeq Ribo Profile (Mammalian) Library Prep Kit index primers 1–12.

### RNA-Seq

CCD18-Co, CaCo2, HCEC-1CT and PBMC cells were cultured and stimulated with either LPS, IL-22, TNFα, IFN-g, IL-1b, OSM or TGFb as previously described.

Messenger RNA was purified from total RNA using poly-T oligo-attached magnetic beads. After fragmentation, the first strand cDNA was synthesized using random hexamer primers, followed by the second strand cDNA synthesis using either dUTP for directional library or dTTP for non-directional library. For the non-directional library, it was ready after end repair, A-tailing, adapter ligation, size selection, amplification, and purification. For the directional library, it was ready after end repair, A-tailing, adapter ligation, size selection, USER enzyme digestion, amplification, and purification. Library preparation used the NEBNext Ultra II RNA Library Prep Kit for Illumina. The library was checked with Qubit and real-time PCR for quantification and bioanalyzer for size distribution detection. Quantified libraries were pooled and sequenced on NovaSeq6000, according to effective library concentration and data amount. The clustering of the index-coded samples was performed according to the manufacturer’s instructions. After cluster generation, the library preparations were sequenced on a NovaSeq6000 and paired-end reads were generated.

### Annotation of novel smORFs

The bioinformatic pipeline used to annotate smORFs is described in detail in Martinez et al. with a few modifications [20]. First, all reads were aligned to the hg38 reference genome using STAR v2.79a [70]. Next, instead of Cufflinks [71], Stringtie v2.2.1 [72] was used to generate total transcriptome form RNA-seq reads, followed by 3-frame translation using a GTFtoFASTA [20]. This generates a ORF database to which we used RibORF v0.1 [46, 73] to score the ribosome profiling reads. The ORF database was filtered using a threshold score of ≥ 0.7, ≤ 150 AA in length, and novel (not overlapping with known coding regions in RefSeq, and a maximum BLASTp alignment e-value to UniProt [74, 75]). The resulting smORF database was then annotated using Bedtools closest function against the hg38 reference genome to determine genome overlap [76]. This also allowed splitting the smORFs into categories based on their genomic location. smORF overlaps between ribosome profiling reads were determined by quantifying read counts using HOMER analyzeRepeats and a count threshold of ≤ 10 was used [51]. Conservation was determined using tBLASTn against the reference genomes of *Pan troglodytes*, *Rattus norvegicus*, *Mus musculus*, *Equus caballus*, *Ovis aries*, *Bos taurus*, *Sus scrofa domesticus*, and *Canis lupis familiaris* with a e-value threshold of ≤ 0.001 [77]. Generation of BigWig files for Ribo-seq read coverage visualization was done using bamCoverage 3.5.1 (Part of the deepTools suites) [78].

PRICE v1.0.4 [48]and Ribocode v1.2.14 [49] were used alongside RibORF to identify microproteins based on the Ribo-Seq data. For PRICE, the trimmed reads without ribosomal contamination were aligned to the genome the same way we did for RibORF. For Ribocode, however, since the tool requires the reads to be aligned to the transcriptome, we ran STAR with the following parameters: *--quantMode TranscriptomeSAM GeneCounts --outFilterType BySJout --alignEndsType EndToEnd*. RibORF does not perform automatic detection of potential ORFs and is limited to a user-input list – in our case, the 3-frame translated transcriptome. PRICE and Ribocode, on the other hand, can identify both annotated and unannotated ORFs based on the CDS in the provided GTF annotation file. Since the GTF output from StringTie containing the reference-guided transcriptome assembly contains only ‘transcript’ and ‘exon’ features, we ran Transdecoder (v5.7.1) (https://github.com/TransDecoder/TransDecoder) to identify the longest ORF in each transcript and append the CDS information to the StringTie assembly. These are used as a starting point for PRICE and Ribocode to identify the 3-nt periodicity, and other ORFs can be identified later on. We then sorted the GTF file and ran *GTFupdate* from the Ribocode package to include ‘gene’ features in the GTF file and format it appropriately for Ribocode. To identify ORFs with Ribocode, we first ran *prepare_transcripts*, specifying the custom StringTie GTF file containing predicted CDS features, followed by *metaplots*, to identify the 3-nt periodicity. Lastly, we ran Ribocode with default parameters and filtered the results to obtain an FDR of 1%. To identify ORFs with PRICE, we first ran *gedi -e IndexGenome -nobowtie - nokallisto -nostar -D* and provided the paths to the genome and custom GTF files from StringTie containing the CDS predicted by TransDecoder. Then, we ran *gedi -e Price* with default parameters. Then, we ran *gedi -e ViewCIT* to generate results files filtered to obtain an FDR of 1%. Finally, we included only sequences shorter than 150 aa and removed annotated microproteins from both the Ribocode and PRICE results based on the SwissProt database.

### Differential expression analysis on RNA-seq

For differential expression analysis of the RNA-seq reads from stimulated cells generated in this study or downloaded RNA-seq data from human IBD samples, raw paired-end FASTQ reads were trimmed using Trim Glore v 0.6.6 with Cutadapt v 3.4 to remove library adapter sequences and low-quality bases (using command “-g 20 -length 20”) [79, 80]. The trimmed reads were then aligned by STAR v2.79a to the hg38 reference genome [70]. Aligned reads were quantified using HOMER analyzeRepeats, with the smORFs appended to the annotated reference GTF file [51]. Differential expression between various conditions were analyzed using DESeq2 and significant smORFs were identified using the threshold of Log2FC ≥ 1 and FDR < 0.05 [52]. Venn diagrams and Volcano plot were generated in R using ggplot2 package. Heatmaps were generated using the NG-CHM package in R [81]. Bar graphs were generated using GraphPad Prism 10.

### Mass spectrometry sample preparation and analysis

For each mass spectrometry sample, four total, 10 × 10 cm plates of confluent Caco-2 human intestinal cells were used. The cells were cultured in DMEM (Corning Cat. No. 10-013-CV) with 10% FBS and MEM non-essential amino acids (Thermo Fisher), One set of 10 plates was left undifferentiated and, at day 7, the plates were washed, lysed and enriched for microproteins as detailed below. Three sets of plates of 10 each were differentiated for 21 days prior to treatment. The media from differentiated cells were removed and replaced with DMEM without FBS and plates incubated overnight in medium alone or medium with sodium taurocholate micelles or fatty acids micelles. Medium was removed and cells were washed twice with PBS. Plates of cells were lysed with 50 mM HCl, 0.05% Triton X-100 and 0.1% b-mercaptoethanol. Two ml extraction buffer was used per 10 cm plate, plates were scraped and lysates appropriately pooled. The lysates were centrifuged at 30 K xg for 30 min at 4 C and the supernatants filtered through 5 μm syringe filters. The filtered samples were enriched for microproteins by binding to BondElut C8 cartridges, 40 μm sorbent, (Agilent Technologies, Santa Clara, CA). Approximately 100 mg sorbent was used per 10 mg total lysate protein. Cartridges were prepared with one column volume methanol and equilibrated with two volumes triethylammonium formate (TEAF) buffer pH 3 before the samples were applied. The cartridges were then washed with two column volumes of TEAF and the microprotein enriched fractions were eluted with acetonitrile: TEAF pH 3 (3:1) and lyophilized using a Speed-Vac concentrator. A Bradford protein assay (BioRad, Hercules, CA) was used to measure protein concentration of each sample after extraction and enrichment.

The samples were precipitated by methanol/ chloroform and redissolved in 8 M urea/100 mM TEAB, pH 8.5. Proteins were reduced with 5 mM tris(2-carboxyethyl)phosphine hydrochloride (TCEP, Sigma-Aldrich) and alkylated with 10 mM chloroacetamide (Sigma-Aldrich). Proteins were digested overnight at 37 ^o^C in 2 M urea/100 mM TEAB, pH 8.5, with trypsin (Promega). Digestion was quenched with formic acid, 5% final concentration.

Digested samples were run on a Thermo Q Exactive. The samples were injected directly onto a 25 cm, 100 μm ID column packed with BEH 1.7 μm C18 resin. Samples were separated at a flow rate of 300 nl/min on a nLC 1000. Buffer A and B were 0.1% formic acid in water and 0.1% formic acid in acetonitrile, respectively. A gradient of 5–25% B over 280 min, an increase to 40% B over 60 min, an increase to 90% B over another 10 min and a hold at 90%B for a final 10 min was used for a total run time of 360 min. The column was re-equilibrated with 15 ul of buffer A prior to the injection of sample. Peptides were eluted directly from the tip of the column and nanosprayed into the mass spectrometer by application of 2.8 kV voltage at the back of the column. The Q Exactive was operated in data dependent mode. Full MS1 scans were collected in the Orbitrap at 70 K resolution with a mass range of 400 to 1800 m/z and an AGC target of 5e^6^. A top 10 method was used for MS/MS with an AGC target of 5e^6^, resolution of 17.5 K, minimum intensity of 5000 and NCE of 25. Maximum fill times were set to 120 ms and 500 ms for MS and MS/MS scans, respectively. Quadrupole isolation at 2 m/z was used, monoisotopic precursor selection was enabled, charge states of 2–7 were selected and dynamic exclusion was used with an exclusion duration of 15 s.

Protein and peptide identification were done with Integrated Proteomics Pipeline – IP2 (Integrated Proteomics Applications). Tandem mass spectra were extracted from raw files using RawConverter [82] and searched with ProLuCID [83] against a nonredundant human Uniprot database appended with IEC smORF sequences. The search space included all fully-tryptic and half-tryptic peptide candidates. Carbamidomethylation on cysteine was considered as a static modification. Data was searched with 50 ppm precursor ion tolerance and 600 ppm fragment ion tolerance. Identified proteins were filtered to using DTASelect [84] and utilizing a target-decoy database search strategy to control the false discovery rate to 1% at the protein level [85]. A minimum of 1 peptide per protein and 1 tryptic end per peptide were required and precursor delta mass cutoff of 10ppm.

### Searching of novel smORFs in published HLA peptidomic samples

Raw MS data from PXD013649 was sourced from PRIDE and included immunoaffinity-purified HLA peptides from HB95 cells, HB145 cells, lung tissue, and lung tumors. These raw files were converted into mzML format using ThermoRawFileParser. Subsequently, mzML files were analyzed to detect smORF-encoded microproteins using the FragPipe pipeline. A default workflow was used to identify 7–25 amino acid peptide fragments in HLA-1 complexes with a peptide false discovery rate (FDR) of less than 0.01. The search database included smORFs, the standard human proteome, and typical contaminants, incorporating reverse sequences for a reverse-decoy strategy.

## Supplementary Information

Supplementary Material 1

Supplementary Material 2

Supplementary Material 3

Supplementary Material 4

Supplementary Material 5

Supplementary Material 6

## Data Availability

Raw and processed Ribosome Profiling and RNA sequencing data used for this study have been deposited at Gene Expression Omnibus database under accession number GSE269376.

## Abbreviations

IBD: Inflammatory bowel disease
IEC: intestinal epithelial cell
IL1β: Interleukin 1 beta
IFNγ: Interferon gamma
LPS: lipopolysaccharide
PBMC: peripheral blood mononuclear cells
Ribo-Seq: Ribosome Profiling
smORF: small Open Reading Frames
TGFβ: Transforming Growth Factor beta
TNFα: Tumor Necrosis Factor alpha
