# Supplementary material for: Microprotein-encoding RNA regulation in cells treated with pro-inflammatory and pro-fibrotic stimuli
Source: BMC Genomics. 2024 Nov 5;25:1034. doi: 10.1186/s12864-024-10948-1 (PMC11536906; doi:10.1186/s12864-024-10948-1)
Supplement: Supplementary file 1 — Supplementary Material 1 [file 12864_2024_10948_MOESM1_ESM.docx]

**Supporting Information for Submission to Genome Biology**

**Title:** Microprotein-Encoding RNA Regulation in Cells Treated with Pro-inflammatory and Pro-Fibrotic Stimuli

**Authors:** Victor J. Pai*^,1,†^ , Calvin Lau^1, †^, Almudena Garcia-Ruiz^1^, Cynthia Donaldson^1^, Joan M. Vaughan^1^, Brendan Miller^1^, Eduardo V. DeSouza^1^, Antonio M. Pinto^2^, Jolene Diedrich^2^, Narender R. Gavva^3^, Shan Yu^3^, Christopher DeBoever^3^, Shane R. Horman*^,3^, and Alan Saghatelian*^,1^

^†^Contributed equally

1 Clayton Foundation Peptide Biology Laboratories, The Salk Institute for Biological Studies, 10010 North Torrey Pines Road, La Jolla, California 92037, United States

2 Mass Spectrometry Core, The Salk Institute for Biological Studies, 10010 North Torrey Pines Road, La Jolla, California 92037, United States

3 Takeda Development Center Americas, Inc., San Diego, CA 92121, USA

*Corresponding authors: [vpai@salk.edu](mailto:vpai@salk.edu), [shane.horman@takeda.com](mailto:shane.horman@takeda.com), and [asaghatelian@salk.edu](mailto:asaghatelian@salk.edu)

**Supplementary Tables**

Supplementary Table S1. List of smORFs using RibORF, PRICE, and Ribocode.

Supplementary Table S2. tBLASTn output searching for IEC smORFs against various species

Supplementary Table S3. Raw DESEQ2 output for all annotated genes and IEC smORFs

Supplementary Table S4. Mass Spectrometry search on Caco2 cells for UNIPROT genes and IEC smORFs. smORFs are highlighted in red.

Supplementary Table S5. Mass Spectrometry search for IEC smORFs against HLA-I proteomic datasets downloaded from PXD013649 (PMID: **32157095)**


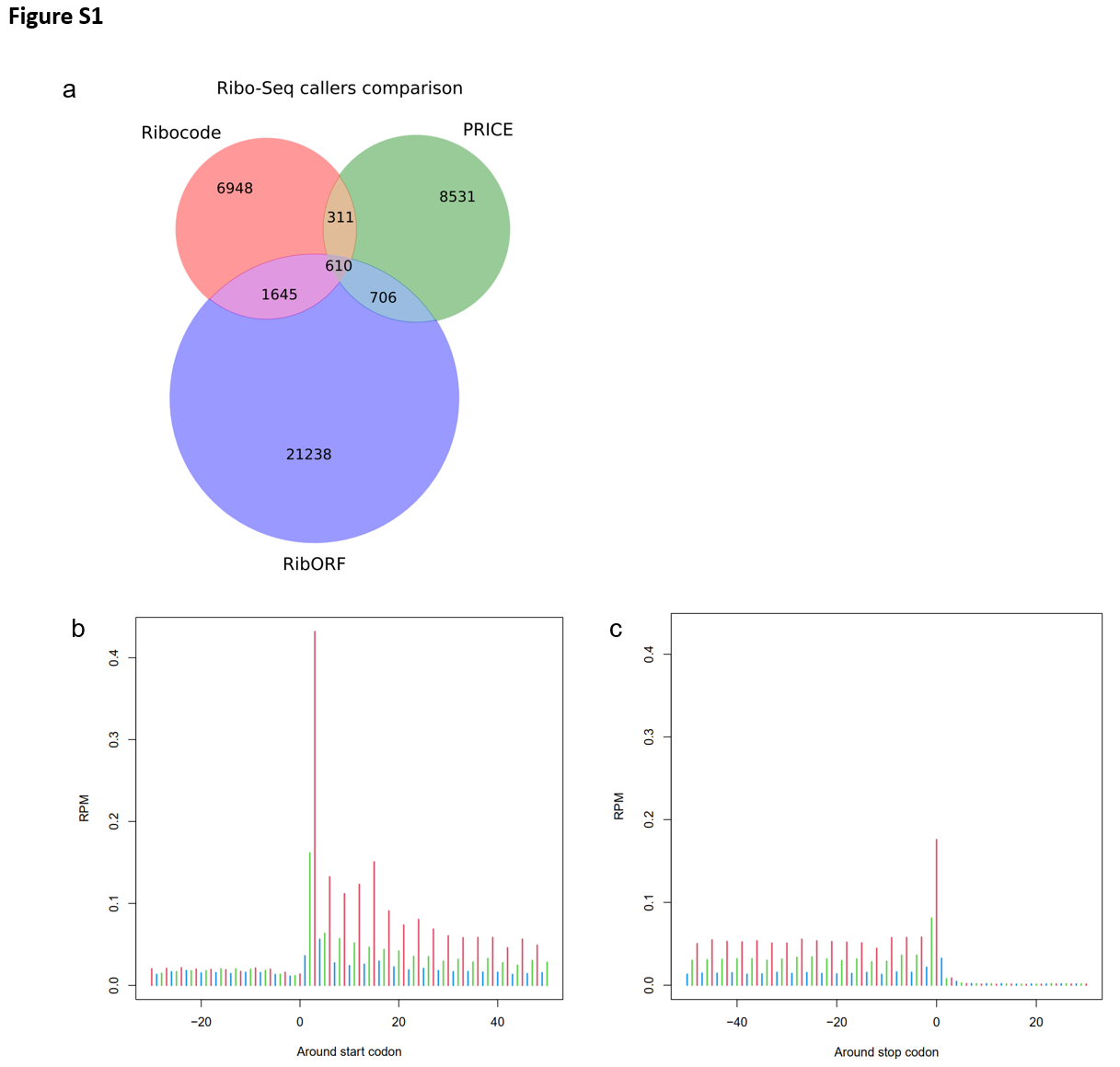

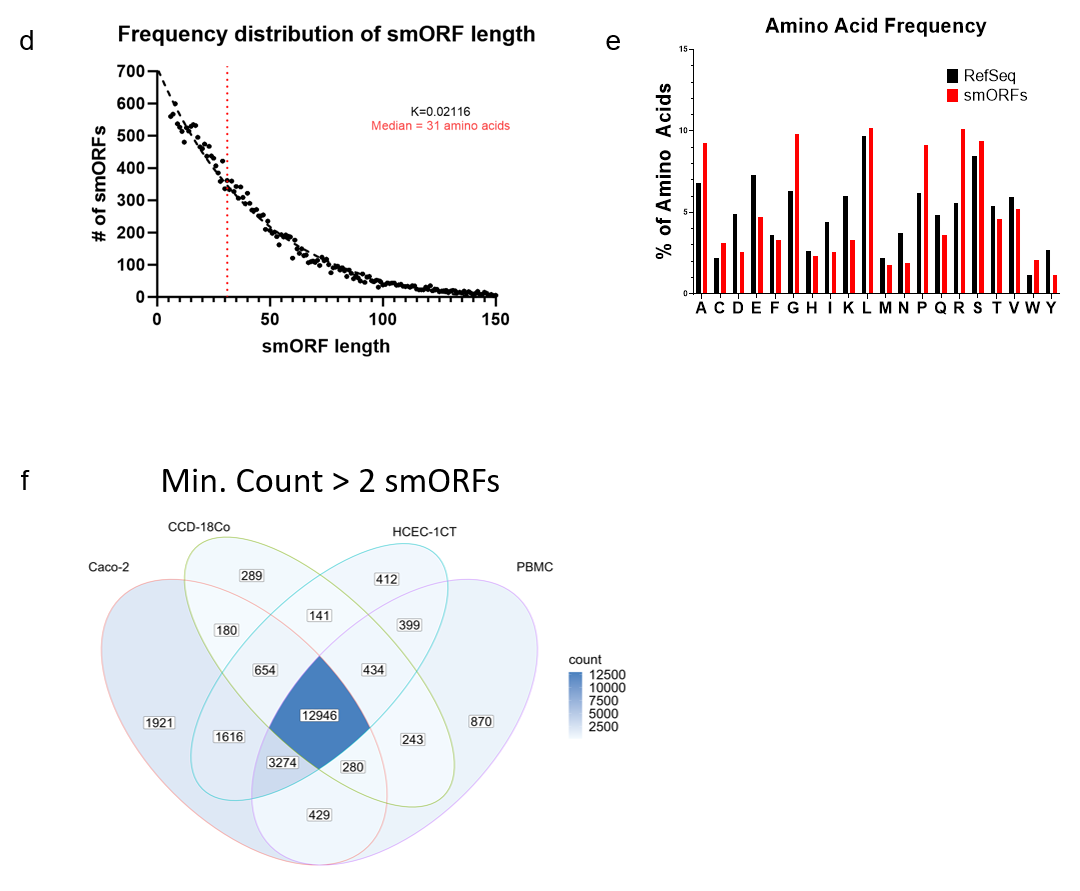


Figure S1. Properties of IEC smORFs.

a) Venn diagram showing overlap of smORFs identified by different Ribosome Profiling callers.

b, c) representative plots showing 3 nucleotide periodicity from ribosome profiling experiment results.

d) Frequency distribution of smORF length. The median length of smORFs is 31 amino acids.

e) Amino acid frequency of all IEC smORFs. In general, the smORFs are enriched in alanine, glycine, proline, and arginine.

f) Venn Diagram showing smORF coverage among different cell line by read counts. Using a minimum read count threshold of 2 per cell line, only 14% of smORFs were found to be specific to each cell line.


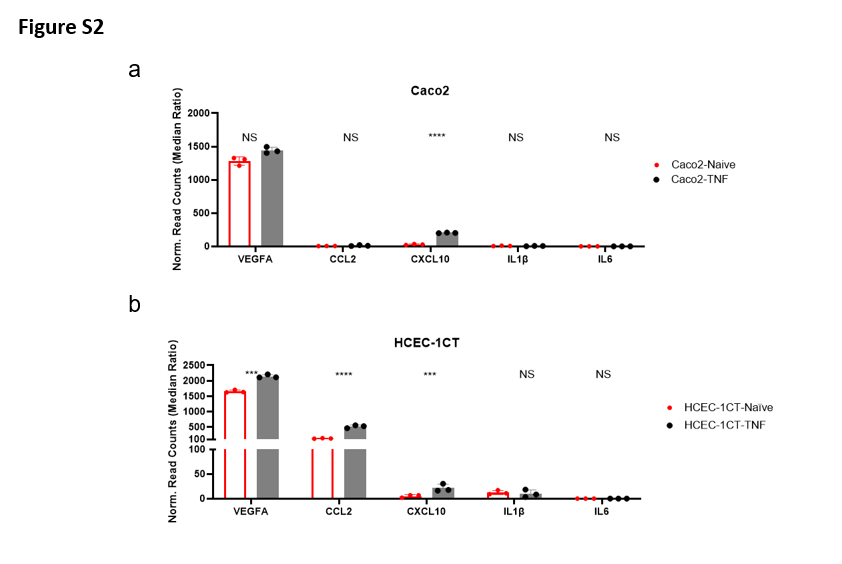


Figure S2. Minimal effect of TNFα treatment Caco2 and HCEC-1ct cells on various inflammatory genes.

1. Bar graph of normalized read counts (by median of ratio from DESeq2) in Caco2 cells treated with vehicle control (naïve cells) versus TNFα
2. Bar graph of normalized read counts (by median of ratio from DESeq2) in HCEC-1CT cells treated with vehicle control (naïve cells) versus TNFα

Differential expression analysis was performed through DESeq2, and comparisons were tested by Wald’s test followed by Benjamini and Hochberg correction. *** = adj. p < 0.001; **** = adj. P < 0.0001; NS = not significant.


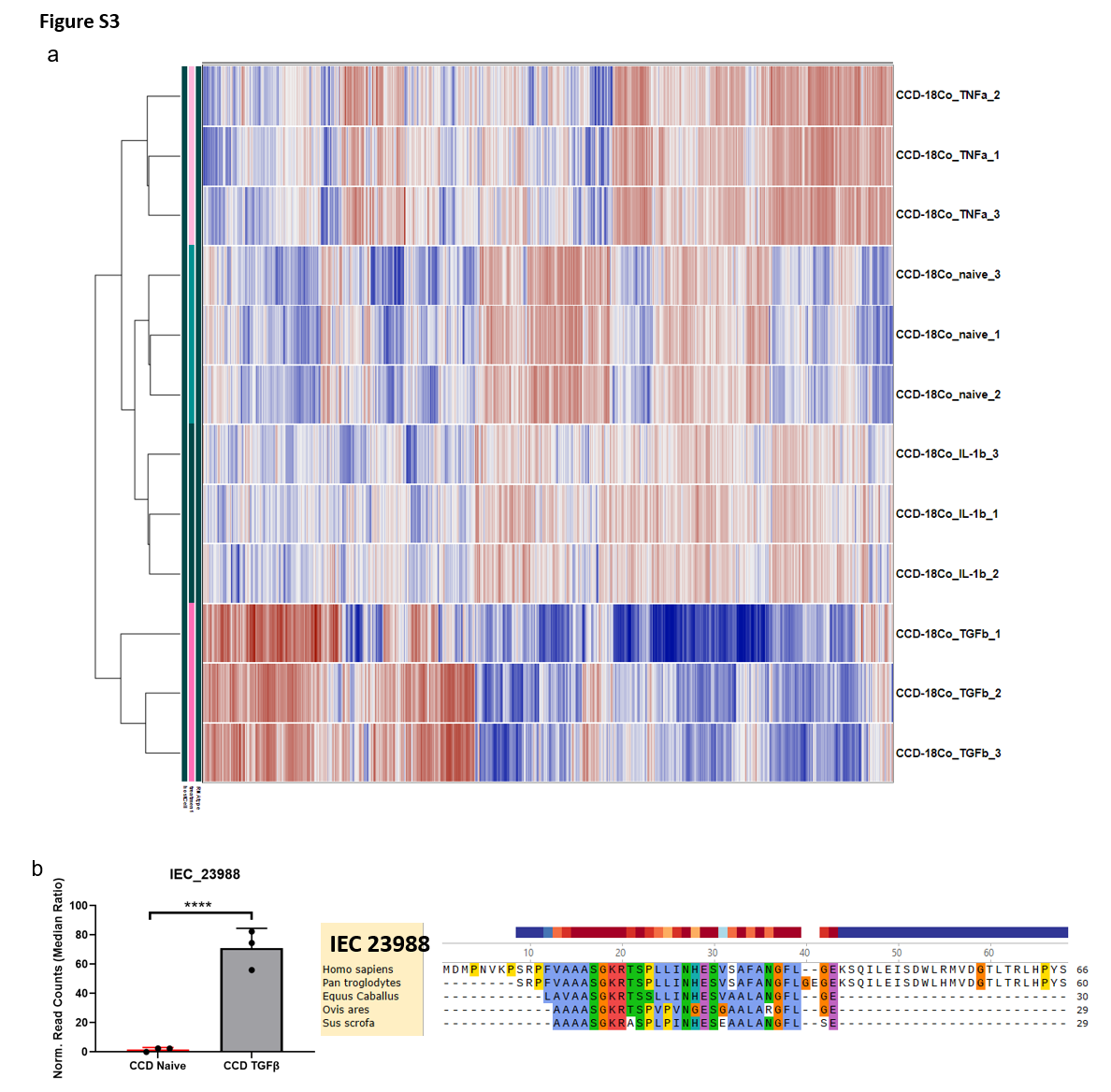


Figure S3. Differential expression analysis smORFs in CCD18-co cells

1. Hierarchical heat map of all differentially expressed smORFs (FDR < 0.05) in CCD-18co cells under various treatments.
2. Upregulation of IEC_23988 in CCD18-co cells after TGFβ treatment. IEC_23988 is a predicted novel protein ENSG00000222032 (chr2:237425953-237434678). Amino acid level conservation (d) show partial match to various species all without the start codon.

Bar graphs (b) showing normalized read counts of smORF. Differential expression analysis was performed through DESeq2, and comparisons were tested by Wald’s test followed by Benjamini and Hochberg correction, **** = FDR < 0.0001

Amino acid level conservation (e) of the predicted smORFs sequences to other species by tBLASTn


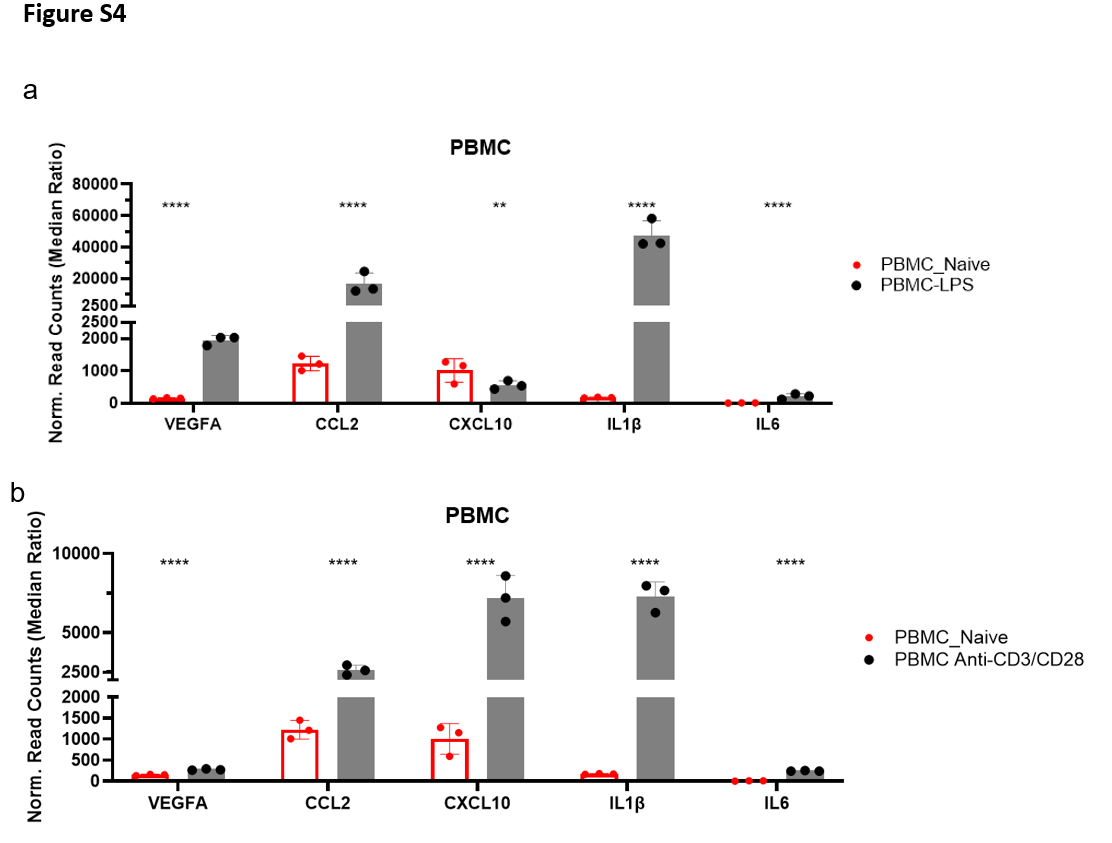

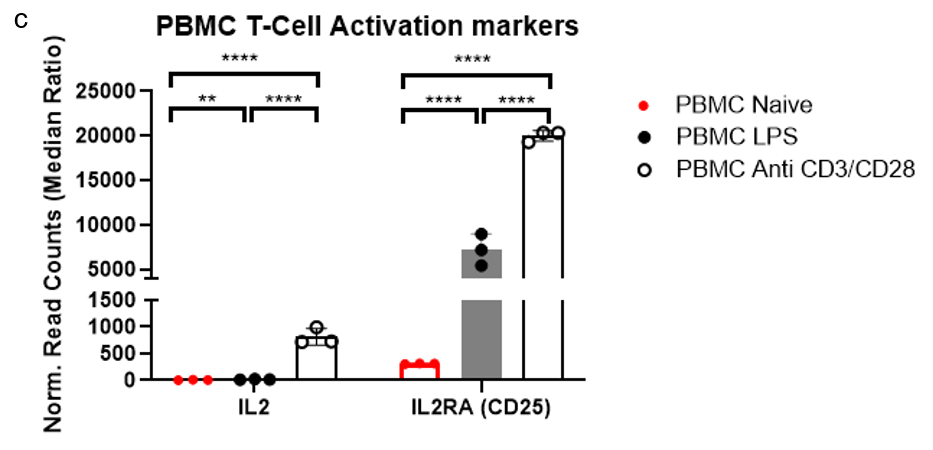

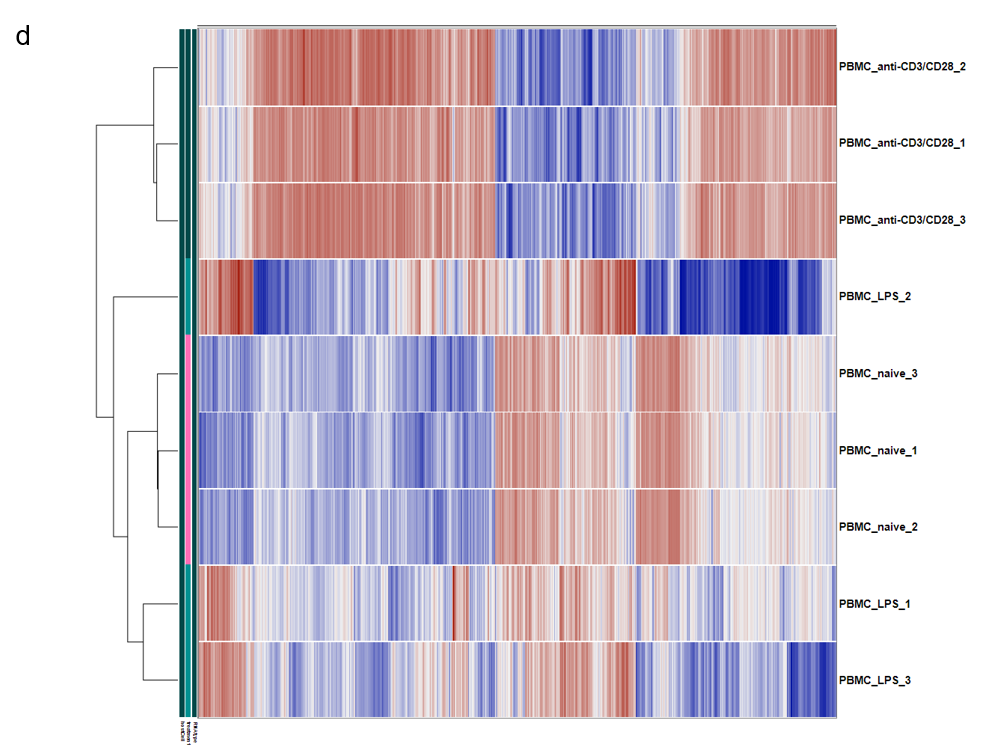


Figure S4. Differential expression analysis on PBMC cells after LPS or Anti-CD3/CD28 treatment

a, b) Bar graph showing normalized read counts of inflammatory markers VEGFA, CCL2, CXCL10, IL1b, and IL6 in PBMC control vs LPS (a), and PBMC vs anti-CD3/CD28 (b).

c) Bar graph showing normalized read counts of T-cell activation markers IL2 and IL2RA (CD25) in PBMCs after LPS or Anti-CD3/CD28

d) Hierarchical heat maps of the all differentially expressed smORFs (FDR < 0.05) in PBMCs treated with LPS, anti-CD3/CD28, and vehicle control (naïve).

Bar graphs (a, b, c) showing normalized read counts of smORF. Differential expression analysis was performed through DESeq2, and comparisons were tested by Wald’s test followed by Benjamini and Hochberg correction, ** = FDR < 0.01, **** = FDR < 0.0001.


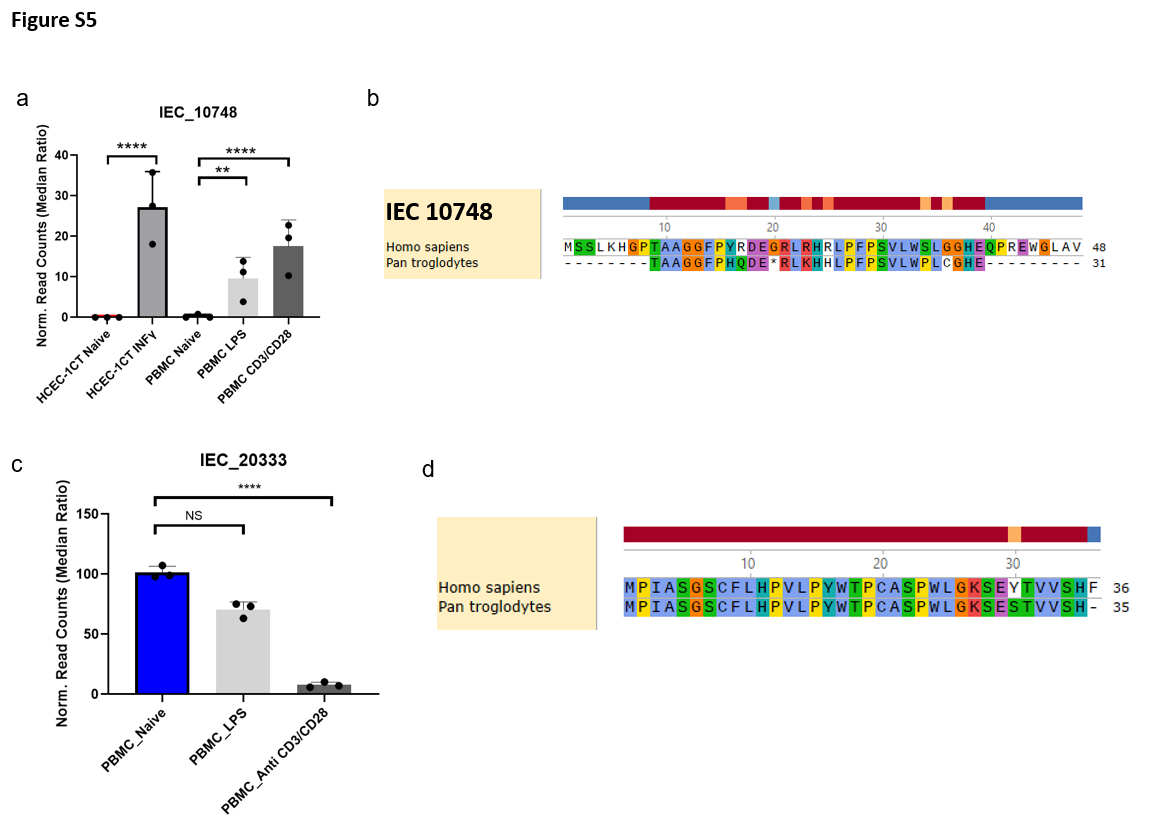

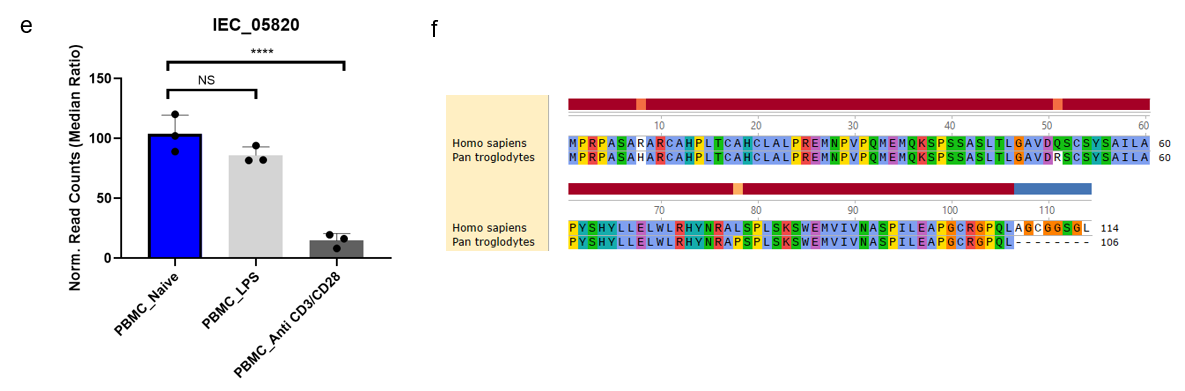


Figure S5. Additional examples of differentially expressed smORFs among IEC cell lines.

(a, b) IEC_10748 is a poorly conserved smORF found on the pseudogene LGALS17A (chr19:39679420-39682126). Amino acid level conservation (b) show a partial match to pan troglodytes but without the start codon.

(c, d) IEC_20333 is a smORF found overlapping the non-coding RNA LINC00861 (chr8:125946171-125951009). Amino acid level conservation (f) show match to pan troglodytes across the whole predicted protein.

(e, f) IEC_05820 is a smORF found overlapping the non-coding RNA LINC00649 (chr21:33931424-33945832). Amino acid level conservation (h) show match to pan troglodytes across the whole predicted protein.

Bar graphs (a, c, e, g) showing normalized read counts of smORF. Differential expression analysis was performed through DESeq2, and comparisons were tested by Wald’s test followed by Benjamini and Hochberg correction, ** = FDR < 0.01 ; **** = FDR < 0.0001


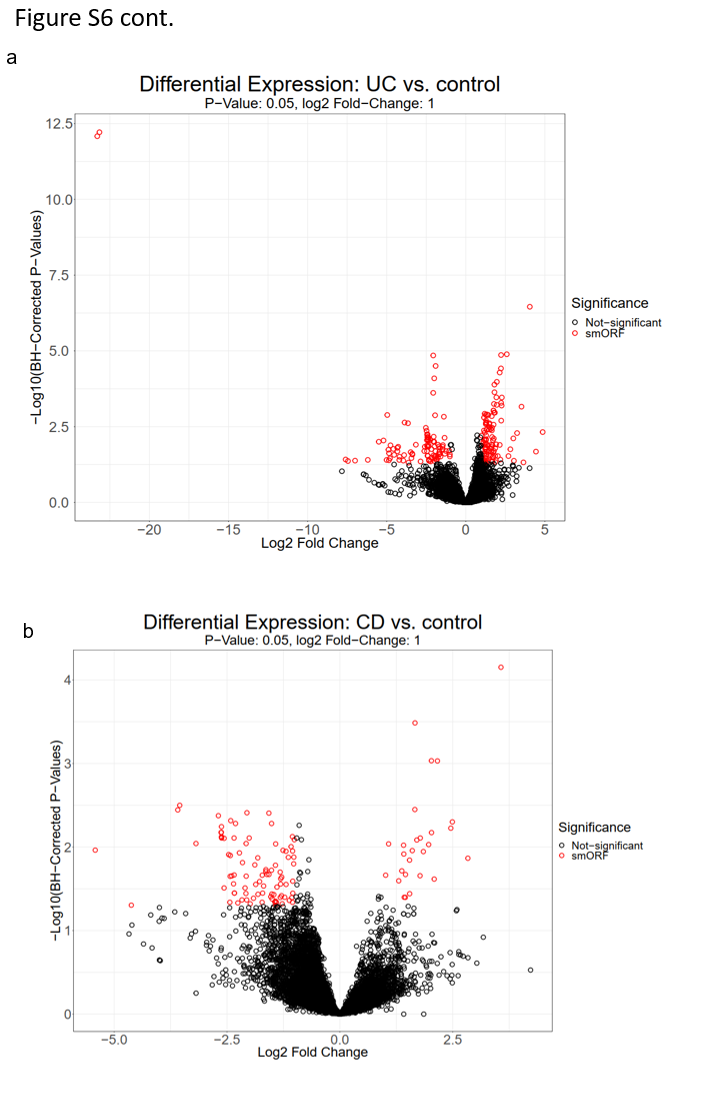


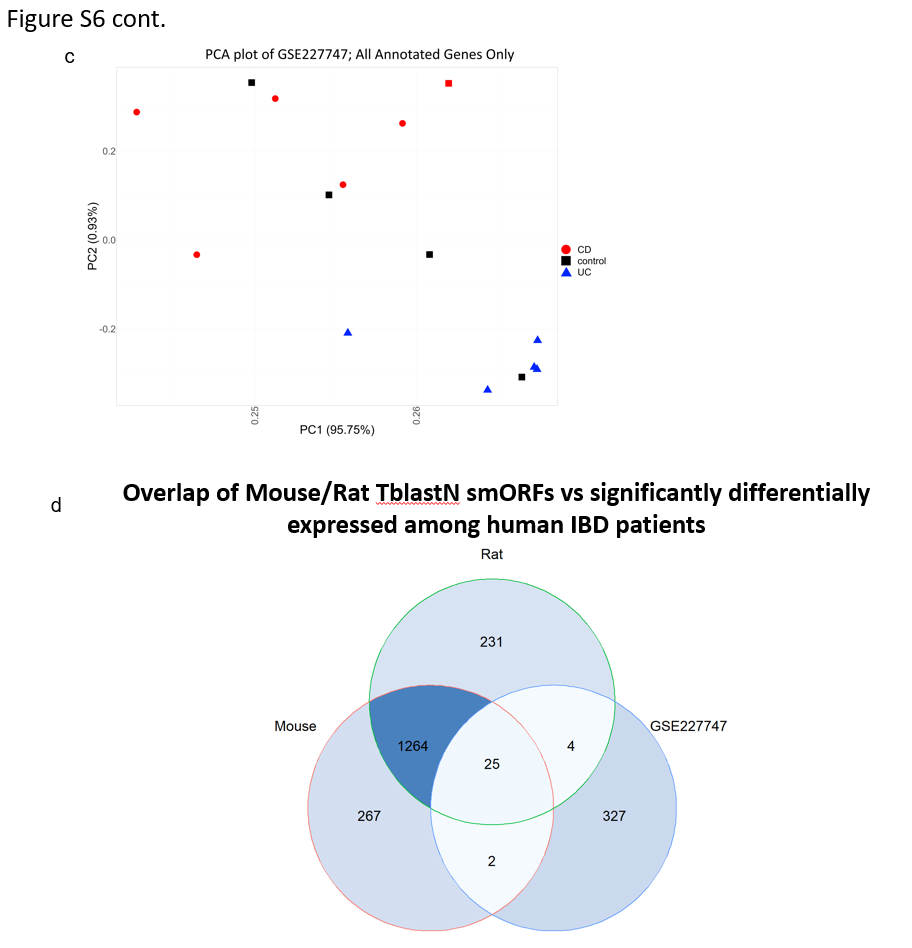


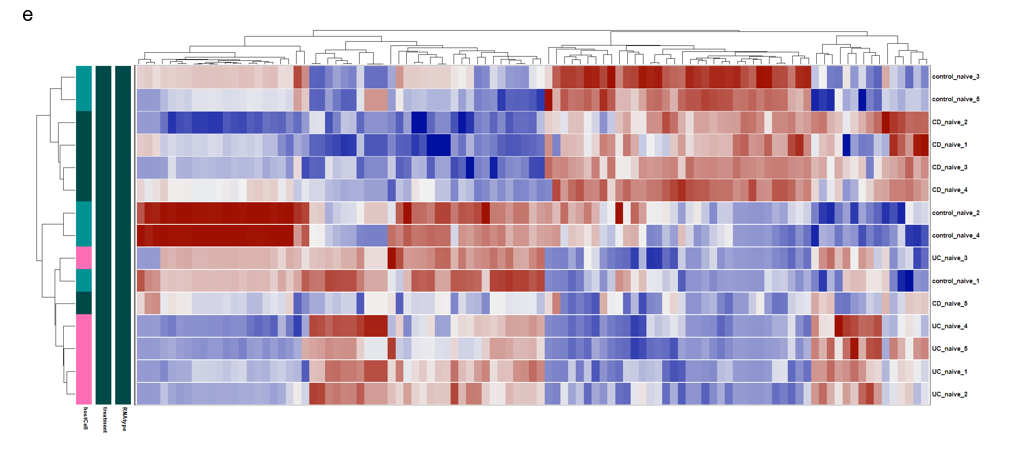


Figure S6 Differential expression analysis of RNA-seq from IBD patient biopsies.

a & b) Volcano plots of differential expression analysis on smORFs between ulcerative colitis vs control (a) and Crohn’s disease vs control (b). Significant smORFs (Log2FC > 1, FDR < 0.05) are shown in red.

1. PCA analysis of all annotated gene expression from human IBD patient biopsies (GSE227747).
2. Overlap of conserved smORFs (mouse/rat, as predicted by tBLASTn), and differentially expressed smORFs from human IBD patients (GSE227747).
3. Hierarchical heatmap analysis of top 40 differentially expressed smORFs from IBD patient biopsies.


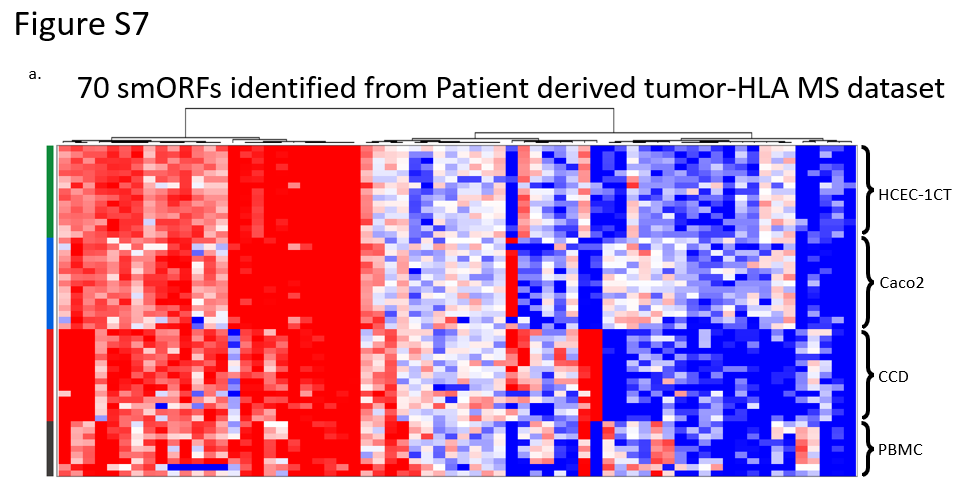


Figure S7. Differential expression analysis of smORFs identified from tumor-HLA mass spectrometry.

1. Hierarchical heatmap analysis of all 70 smORFs identified from patient derived tumor-HLA MS dataset (PMID: **32157095).**
